# The gastrointestinal antibiotic resistome in pediatric leukemia and lymphoma patients

**DOI:** 10.3389/fcimb.2023.1102501

**Published:** 2023-02-24

**Authors:** Tamara MacDonald, Katherine A. Dunn, Jane MacDonald, Morgan G.I. Langille, Johan E. Van Limbergen, Joseph P. Bielawski, Ketan Kulkarni

**Affiliations:** ^1^ Department of Pharmacy, IWK Health, Halifax, NS, Canada; ^2^ Faculty of Health Professions, Dalhousie University, Halifax, NS, Canada; ^3^ Department of Pediatrics, Division of Hematology Oncology, Izaak Walton Killam (IWK) Health, Halifax, NS, Canada; ^4^ Department of Biology, Dalhousie University, Halifax, NS, Canada; ^5^ Institute for Comparative Genomics, Dalhousie University, Halifax, NS, Canada; ^6^ Department of Science, University of Waterloo, Waterloo, ON, Canada; ^7^ Department of Pharmacology, Faculty of Medicine, Dalhousie University, Halifax, NS, Canada; ^8^ Department of Pediatric Gastroenterology and Nutrition, Emma Children’s Hospital, Amsterdam University Medical Centers, Amsterdam, Netherlands; ^9^ Tytgat Institute for Liver and Intestinal Research, Amsterdam Gastroenterology Endocrinology and Metabolism, Amsterdam University Medical Centers, University of Amsterdam, Amsterdam, Netherlands; ^10^ Department of Mathematics & Statistics, Dalhousie University, Halifax, NS, Canada

**Keywords:** antibiotic resistance genes, resistome, leukemia, lymphoma, pediatric, microbiome

## Abstract

**Introduction:**

Most children with leukemia and lymphoma experience febrile neutropenia. These are treated with empiric antibiotics that include β-lactams and/or vancomycin. These are often administered for extended periods, and the effect on the resistome is unknown.

**Methods:**

We examined the impact of repeated courses and duration of antibiotic use on the resistome of 39 pediatric leukemia and lymphoma patients. Shotgun metagenome sequences from 127 stool samples of pediatric oncology patients were examined for abundance of antibiotic resistance genes (ARGs) in each sample. Abundances were grouped by repeated courses (no antibiotics, 1-2 courses, 3+ courses) and duration (no use, short duration, long and/or mixed durationg) of β-lactams, vancomycin and “any antibiotic” use. We assessed changes in both taxonomic composition and prevalence of ARGs among these groups.

**Results:**

We found that Bacteroidetes taxa and β-lactam resistance genes decreased, while opportunistic Firmicutes and Proteobacteria taxa, along with multidrug resistance genes, increased with repeated courses and/or duration of antibiotics. Efflux pump related genes predominated (92%) among the increased multidrug genes. While we found β-lactam ARGs present in the resistome, the taxa that appear to contain them were kept in check by antibiotic treatment. Multidrug ARGs, mostly efflux pumps or regulators of efflux pump genes, were associated with opportunistic pathogens, and both increased in the resistome with repeated antibiotic use and/or increased duration.

**Conclusions:**

Given the strong association between opportunistic pathogens and multidrug-related efflux pumps, we suggest that drug efflux capacity might allow the opportunistic pathogens to persist or increase despite repeated courses and/or duration of antibiotics. While drug efflux is the most direct explanation, other mechanisms that enhance the ability of opportunistic pathogens to handle environmental stress, or other aspects of the treatment environment, could also contribute to their ability to flourish within the gut during treatment. Persistence of opportunistic pathogens in an already dysbiotic and weakened gastrointestinal tract could increase the likelihood of life-threatening blood borne infections. Of the 39 patients, 59% experienced at least one gastrointestinal or blood infection and 60% of bacteremia’s were bacteria found in stool samples. Antimicrobial stewardship and appropriate use and duration of antibiotics could help reduce morbidity and mortality in this vulnerable population.

## 1 Introduction

Immunosuppressive treatments confer an increased risk of infectious complications in children with leukemia and lymphoma (Morrison, 2014; Logan et al., 2020). To reduce infection related morbidity and mortality broad-spectrum antipseudomonal β-lactams, vancomycin and aminoglycosides are given frequently and often for extended periods of time (Lehrnbecher et al., 2017), which can result in dysbiosis or an “imbalance” in the gastrointestinal (GI) microbiome (Dethlefsen et al., 2008; Dethlefsen and Relman, 2011; Vangay et al., 2015; Iizumi et al., 2017). Children with leukemia and lymphoma are also subjected to high doses of chemotherapy over a period of many months to years and this can affect the integrity of the gastrointestinal tract (GIT) barrier (Keefe et al., 2000; Duncan and Grant, 2003; Wang et al., 2013). The loss of GIT integrity increases the chance that members of the dysbiotic GIT microbial community can cross the GI barrier, including those with antibiotic resistance genes (ARGs) (Sonis, 2004; Stringer et al., 2009; Huddleston, 2014). Bacteremia can lead to sepsis, and the presence of ARGs in these bacteria increases the risk of death (De Oliveira et al., 2020).

The GIT is colonized by large numbers of microorganisms, including bacteria, viruses and fungi, with Firmicutes and Bacteroidetes being the main bacterial phyla (Arumugam et al., 2011). At diagnosis, children with acute lymphoblastic leukemia (ALL) have been characterized with reduced microbiome diversity (Bai et al., 2017; Liu et al., 2020; Oldenburg et al., 2021). Bacterial diversity also declines with antibiotic use, however, the actual taxonomic change depends on the class of antibiotic used, along with number of doses, duration of use and mechanism of action (Dethlefsen et al., 2008; Dethlefsen and Relman, 2011; Iizumi et al., 2017). Antibiotic overuse can also result in an increase in taxa that carry antibiotic resistance genes (ARGs) in the microbial resistome.

The microbial resistome is the collection of all ARGs and their precursors in a multi-species community of pathogenic and nonpathogenic bacteria (Kim and Cha, 2021). ARGs are bacterial genes that enable the bacteria to generate a mechanism to prevent its death from an antibiotic. ARGs within a species can be innate (i.e., a fixed component of the species core genome) or polymorphic (i.e., opportunistically acquired by only some lineages *via* a gene transfer event). Bacteria can carry ARGs specific to a single antibiotic or to multiple antibiotics (Nielsen et al., 2021). β-lactam exposure was shown to result in selection for pathogenic Enterobacteriaceae, including *Escherichia, Klebsiella, and Enterobacter* with resistance genes (Vázquez-López et al., 2021). *Enterococcus*, a nosocomial Firmicutes pathogen, both naturally contains ARGs and can quickly acquire ARGs including multidrug resistance genes resulting in resistance to vancomycin as well as to other antimicrobial agents (Ahmed and Baptiste, 2018). Furthermore, any increase in bacteria that harbor ARGs will increase the pool of ARGs in the resistome, which will increase the difficulty of treatment (Nielsen et al., 2021).

Chemotherapy negatively impacts the integrity of the gut epithelium (due to decreased cell turnover), and directly effects the microbiome (Oldenburg et al., 2021). Common agents administered to children with leukemia and lymphoma include methotrexate which reduce abundance of commensal bacteria (Huang et al., 2012) and corticosteroids which increase abundance of certain pathogenic taxa (Franzin et al., 2021). In addition, cyclophosphamide leads to increased intestinal permeability which can result in translocation of pathogenic species (Yang et al., 2013). More broadly, the decline in bacterial diversity that follows chemotherapy treatment (Rajagopala et al., 2016; Hakim et al., 2018; De Pietri et al., 2020), can predispose the immunocompromised host to pathogenic species. We expect that the interaction of changes to gut epithelium and microbiome composition during chemotherapy may involve unique effects (dysbiosis) that can’t be predicted from the historical studies of microbiome changes in patients not on chemotherapy.

Children with leukemia and lymphoma are treated with multiple antibiotics, which can change bacterial diversity, increase the overall frequency of ARGs in the GIT, and change distribution of ARGs within both pathogens and non-pathogens. The pioneering work of Margolis and coauthors (2021) examined the impact of prophylactic levofloxacin on the resistome during leukemia induction. They found that the use of prophylactic levofloxacin increased the prevalence of ARGs specific to this antibiotic. However, their study did not address (i) other antibiotics routinely used in leukemia and lymphoma patients, (ii) the entirety of the treatment period or (iii) the impact of repeated courses or duration of treatment. A better understanding of the prevalence of ARGs in the GIT resistome, and its dynamics during antibiotic treatment, will have direct relevance to the choice and duration of optimal antibiotic treatment and inform antimicrobial stewardship programs (Muratore et al., 2022). Currently, the impact of antibiotic use on the resistome in this vulnerable pediatric oncology population is not well understood. However, whole shotgun metagenomic sequencing can be used to identify the bacteria and ARGs present in the GIT resistome. In this study we employed metagenomic sequence data to investigate the impact of repeated courses of antibiotics on the relative abundance of ARGs. Given that the mix and duration of antibiotic treatment varies among institutions, we directly tested an important, and as yet untested, hypothesis; does an increased number of courses and longer duration of antibiotic treatment increase the prevalence of resistance genes specific to the antibiotics administered in children with leukemia/lymphoma.

## 2 Methods

### 2.1 Patient recruitment and sample collection

We investigated 39 pediatric patients with either leukemia (33) or lymphoma (6) from the Atlantic Maritime provinces of Canada, undergoing care at the IWK Health Centre in Nova Scotia, Canada (**Table 1**). The 33 leukemia patients included 28 acute lymphoblastic leukemia (ALL) patients and five acute myeloid leukemia (AML) patients, and the six lymphoma patients included two hodgkin lymphoma (HL) patients and four non-hodgkin lymphoma (NHL) patients. Specific on subtypes of cancers and treatment protocols for each cancer type are included in **Supplemental Material**. Patients were asked for stool samples prior to start of chemotherapy and based on convenience while in hospital for treatment, however given the difficulties of collection in this patient population and variable assistance of clinical staff there was no prescribed pattern to sample collection and number of samples and timing varied considerably between patients. Timelines of stool sample collection are in supplemental material (**Supplementary Figure S1**). A total of 127 stool samples were collected among the 39 patients with a mean of 3 samples per patient (min=1; max=12; and median=2). Pre-chemotherapy samples were collected for 16 patients (8 ALL; 5 AML; 2 HL; 1 NHL) with 12 of these (6 ALL; 5 AML; 1 HL) also having a sample collected during treatment. Among ALL patients 3.4 samples were collected on average from each patient (median=2; min=1; max=12), AML patients had on average 4.6 samples per patient (median=2; min=1; max=11), HL and NHL patients had an average and median of 1.5 samples per patient (min=1; max=2). Stool samples were stored at -20 C for transport and then frozen at -80 C until analysis. Information on antibiotics given, including length of use and number of courses, were collected from each patient. Stool samples were grouped based on the number and duration of courses received prior to the stool sample. All patients receive trimethoprim-sulfamethoxazole throughout treatment prophylactically and so this antibiotic is not included as part of the use and duration. The two main antibiotics used in this patient population are piperacillin-tazobactam (a β-lactam antibiotic) and vancomycin (a glycopeptide antibiotic), although piperacillin-tazobactam is the main β-lactam antibiotic used additional β-lactam antibiotics as well as other antibiotic classes were also given (**Supplementary Table S1**). Antibiotics were given either intravenously or orally and method of administration is noted in **Supplemental Table S1**. Samples were grouped based on multiple-use of: 1) β-lactams, 2) vancomycin, and 3) “any antibiotic” use (AAb). The defined groups within each were: no use (except for trimethoprim-sulfamethoxazole), 1-2 courses, and 3+ courses, of the above 3 groups of antibiotic(s). Additionally, the number of courses of β-lactams, vancomycin, and AAb given were also examined. Samples were also grouped based on duration of 1) β-lactams, and 2) vancomycin. The three defined groups within these two treatments were: no use (except for trimethoprim-sulfamethoxazole), short duration (prescribed ≤10 days), and long (prescribed >10 days) or mixed duration (mix of short and long courses) referred to as long/mixed (LM). Additionally, the number of days of β-lactam and vancomycin antibiotics given were also examined. Timelines of stool sample collection and antibiotic use are in supplemental material (**Supplementary Figure S1**). The IWK Health Centre Research Ethics Board approved the study and all patients and/or their guardians provided informed consent or assent.

**Table 1:** Patient and stool sample information.

	no use	1-2 courses	3+ courses	short duration ^a^	LM duration ^b^	p value ^c^
*Patient information*						
β-lactam						
Number of patients	8	31	15	29	14	
Males (%)^d^	4 (50%)	19 (61%)	7 (47%)	19 (66%)	7 (50%)	0.61, 0.54
Age: Over 3 (%)^e^	6 (75%)	24 (77%)	9 (60%)	24 (83)	8 (57%)	0.46, 0.20
Vancomycin						
Number of patients	28	17	9	17	5	
Males (%)^d^	15 (54%)	8 (47)	4 (44%)	9 (53%)	2 (40%)	0.86, 0.85
Age: Over 3 (%)^e^	22 (79%)	11 (65%)	5 (56%)	11 (65%)	3 (60%)	0.35, 0.50
AAb ^f^						
Number of patients	8	27	20	NA	NA	
Males (%)^d^	4 (50%)	17 (63%)	11 (55%)	NA	NA	0.76
Age: Over 3 (%)^e^	6 (75%)	22 (81%)	13 (65%)	NA	NA	0.44
*Stool sample information*						
β-lactam						
Number of stool samples^g^	10(8)	80(31)	37(15)	71(29)	46(14)	
Treatment Phase^g^						
Pre-chemotherapy Induction Post-induction-6month Beyond 6 months	5(5) 5(5) 0 0	12(11) 33(25) 19(12) 16(6)	0 2(2) 25(10) 10(8)	11(10) 26(20) 19(11) 15(6)	1(1) 9(6) 25(8) 11(8)	<.001, <.001
Days from chemotherapy^h^	3 (-4, 18)	69 (-5, 371)	150 (9, 471)	76 (-5, 371)	125 (0, 471)	
Days of β-lactams^i^	0	10 (1, 54)	58 (13, 145)	8.5 (1, 35)	52 (4, 145)	
Days of vancomycin^j^	0	2.5 (0, 39)	20 (0, 48)	1.2 (0, 19)	19 (0, 48)	
Vancomycin						
Number of stool samples^g^	59(28)	46(17)	22(9)	54(17)	14(5)	
Treatment Phase^g^						
Pre-chemotherapy Induction Post-induction-6month Beyond 6 months	13(12) 25(20) 12(7) 9(4)	4(3) 15(12) 19(10) 8(2)	0 0 13(5) 9(7)	4(3) 14(11) 22(9) 14(7)	0 1(1) 10(3) 3(3)	<.001, <.001
Days from chemotherapy^h^	55 (-5, 320)	82 (-4, 371)	185 (73, 471)	111 (-4, 430)	132 (24, 471)	
Days of β-lactams^i^	7 (0, 35)	18(3, 54)	79 (23, 145)	28 (3, 145)	76 (29,1 39)	
Days of vancomycin^j^	0	6.5 (1, 39)	29 (7, 48)	9 (1, 34)	30 (12, 48)	
AAb ^i^						
Number of stool samples^g^	10(8)	61(27)	56(20)	NA	NA	
Treatment Phase^g^						
Pre-chemotherapy Induction Post-induction-6month Beyond 6 months	5(5) 5(5) 0 0	12(11) 23(17) 12(7) 14(5)	0 12(10) 32(12) 12(9)			<.001
Days from chemotherapy^h^	3 (-4, 18)	71 (-5, 371)	121 (5, 471)			
Days of β-lactams^i^	0	7 (1, 23)	46 (4, 145)			
Days of vancomycin^j^	0	0.6 (0, 4)	16 (0, 48)			

a Courses of antibiotic were given for ≤10 days.

b LM indicates long or mixed duration, courses of antibiotic were given for >10 days or a mixture of >10 days and ≤10 days in length.

c p-values calculated using Pearson’s Chi-square, for sex and age, and Fisher’s exact test for phase of treatment.

d Number of male patients and percentage in parentheses.

e Number of patients over 3 years of age and percentage in parentheses.

f AAb includes all antibiotics given (excluding trimethoprim-sulfamethoxazole).

g Number of patients represented by the samples are shown in parentheses.

h Mean number of days from start of chemotherapy treatment that stool sample was collected, with minimum and maximum in parentheses.

i Mean number of days of β-lactams antibiotics given prior to stool sample collection, with minimum and maximum in parentheses.

j Mean number of days of vancomycin antibiotics given prior to stool sample collection, with minimum and maximum in parentheses. NA indicates not applicable as analyses were not done for this comparison.

### 2.2 Pediatric oncology ARG database creation

Quality controlled and decontaminated paired-end shotgun metagenomic sequences from a set of 48 matched oral and stool samples from 24 pediatric oncology patients of varying cancer types (see **Supplementary Table S2**) and 5 healthy relatives were assembled using MEGAHIT (kmer values: 21,29,39,59,79,99,119,141; Li et al., 2015) to create a database of contiguous nucleotide sequences. Protein coding sequences were identified in all contiguous sequences greater than 1000 nucleotides in length using PROKKA (version 1.14.6 Seemann, 2014). All protein-coding sequences were compared to the comprehensive antibiotic resistance database (CARD 2021, McArthur et al., 2013) using resistance gene identifier (RGI 4.2.2) with DIAMOND (Buchfink et al., 2015) for alignment with loose hits included. Nucleotide sequences from all identified matches to ARGs were compiled and used to create a bowtie2 pediatric-oncology-ARG-database using bowtie2-build (Langmead and Salzberg, 2012).

### 2.3 Sequence extraction, metagenomic sequencing, and processing

DNA was extracted from 127 stool samples using Norgen stool DNA isolation kits as per manufacturer’s protocol. A minimum of one nanogram of extracted DNA was prepared using the Nextera XT library preparation kit (Illumina) following manufacturer’s protocol. Libraries were pooled and sequenced using paired end NextSeq sequencing (150 bp) (Illumina). The Microbiome Helper metagenomic standard operating procedure was followed (https://github.com/LangilleLab/microbiome_helper/wiki/Metagenomics-standard-operating-procedure-v2). Paired forward and reverse reads were run through the kneaddata pipeline for sequence pre-processing. Low quality sequences (reads smaller than 50 base pairs, and with low quality scores PHRED <Q20) were removed using Trimmomatic (Bolger et al., 2014). Bowtie2 (Langmead and Salzberg, 2012) implemented through kneaddata was used to screen out human and PhiX174 contaminant sequences. High-quality, decontaminated forward and reverse paired sequences were concatenated into a single FASTQ file and METAPHLAn3 (Beghini et al., 2021) was used to profile the taxonomic composition of the sequences in each sample. Only taxa present in ≥10% of samples were included in analyses.

### 2.4 Mapping sample sequence reads to pediatric-oncology-ARG-database

The quality-controlled, decontaminated forward and reverse paired sequences from the 127 leukemia and lymphoma samples were mapped to the pediatric-oncology-ARG-database created using bowtie2 (Langmead and Salzberg, 2012). Counts of sequence reads that mapped to each ARG in the database were obtained for each sample using samtools “sort”, “index” and “idxstat”. Mapped read counts were corrected by the number of sequence reads in each sample. While reads were mapped to all ARG sequences identified, only those ≥60% sequence identity were used in downstream analyses. Antibiotic classes were assigned to each ARGs using the CARD database designation, with two exceptions, 1) genes that occurred in an antibiotic class connected with β-lactam drugs were coded as β-lactam antibiotic class genes (*i.e*., carbapenem, penam, *etc*.), 2) genes that occurred in multiple antibiotic classes (*i.e*., penam, fluoroquinolone, glycopeptide), were coded as “multidrug” antibiotic class genes. Counts within samples assigned to the same gene were summed for downstream analysis. Only genes present in ≥5% of samples were used. Genes in four antibiotic classes were selected for closer analysis: β-lactam antibiotic class, glycopeptide antibiotic class, peptide antibiotic class, and multidrug antibiotic class. These classes were specifically selected as the β-lactam antibiotic class and multidrug antibiotic class potentially contains genes for resistance to β-lactam antibiotics, and the glycopeptide antibiotic class, peptide antibiotic class (a parent class to glycopeptide antibiotics), and multidrug antibiotic class potentially contains genes for resistance to vancomycin. All analyses were carried out on gene sequence data, no allele or SNP information was used.

### 2.5 Statistical analysis

The diversity of species in a community depends on both the number of species observed (species richness) and the relative abundance of species in the community (species evenness). Two communities can have similar numbers of species, but in one community a single species could dominate resulting in differences in evenness between communities. ANOVA and *post hoc* Tukey’s test was employed to examine changes in richness and evenness in samples with repeated courses (no use, 1-2 courses, 3+ courses of β-lactam, vancomycin, AAb) and increased duration (no use, short, LM of β-lactams and vancomycin) of antibiotics.

Sampling varied from patient to patient and consequently some patients were sampled more than others. Patient samples could differ for phase of chemotherapy, and number and duration of antibiotics. Samples were aggregated over patients and combined into the groups defined above (e.g., number of courses or duration). Note that variation in sampling among groups was *not* caused by non-random selection or removal of patients. Nevertheless, we wanted to ensure that the groups did not contain a large number of samples originating from just one patient. Hence, we tested for uniform contribution of patients to the samples within a group *via* the G-test (*α*<0.05) as implemented in the DescTools R package (Signorell et al., 2022). In each group where the uniform contribution was rejected, we carried out a backward-elimination procedure at the patient level. Specifically, the patient with the largest number of samples was removed (i.e., all samples for that patient were removed only for the group being tested) and the test was repeated for that group. This procedure was repeated until the G-test was no longer significant. In this way patients with a potential overrepresentation to a particular group were identified and removed. While this does avoid the potential for a single patient driving the results, it is expected to lower efficiency in the case where data are missing at random.

Differential abundance analysis of taxa abundance based on number of courses (1-2 vs 3+) of β-lactams, vancomycin and AAb, and duration of courses (short vs LM) of β-lactam and vancomycin were investigated using ANOVA-like differential expression (ALDEx2) (Fernandes et al., 2013). ALDEx2 uses center log ratio transformation and the inter-quantile log ratio (IQLR) was used to determine the features to retain as the denominator of the geometric mean calculation. Using IQLR, accounts for data with systematic variation and centers on the set of taxa that have a variance that is between the lower and upper quartile of variance. The method calculates the expected false discovery rate given biological and sampling variation based on a Wilcoxon Rank Sum test using Benjamini Hochberg (BH) (Benjamini and Hochberg, 1995) (α_BH_<0.05).

Differences in mean abundance of the ARGs in samples was examined under three scenarios based on multiple courses of: 1) β-lactam antibiotics, 2) vancomycin antibiotic and 3) “any antibiotic” (AAb) prior to sampling. Mean abundance of each ARG was compared between groups (no use, 1-2 courses, 3+ courses) based on multiple-courses. In addition, abundance of ARGs were compared to number of courses of β-lactam, vancomycin and “any antibiotic” (AAb) prior to sampling. Differences in mean abundance of the ARGs in samples was also examined under two scenarios based on duration of courses of: 1) β-lactam antibiotics, and 2) vancomycin antibiotic prior to sampling. The duration of a course of AAb was not assessed, as the duration was nearly entirely governed by β-lactam antibiotic use. Mean abundance of each ARG was compared between groups (no use, short, LM) based on duration. In addition, abundance of ARGs were compared to number of days of β-lactam, and vancomycin antibiotics prior to sampling. Multivariate linear models using LIMMA (Ritchie et al., 2015) was applied to mean centered, log transformed gene data for all analyses. To account for differences in treatment between cancers and the effect of time in treatment we included cancer diagnosis, and days from start of chemotherapy as confounders. Results were corrected for multiple tests using BH (α_BH_<0.05). For the purpose of discovering candidate ARGs and generating novel hypotheses we employ α_BH_<0.1 (Mallick et al., 2021).

Genes identified as significant belonging to the β-lactam, glycopeptide, peptide and multidrug antibiotic classes were examined for correlation with taxa present in samples using spearman correlation implemented using corr.test in the psych R package version 2.1.9 (Revelle, 2021). Significance was corrected for multiple tests using BH (Benjamini and Hochberg, 1995) (α_BH_<0.05).

## 3 Results

### 3.1 Patient and sample information

Among the 39 pediatric patients, 28 had acute lymphoblastic leukemia (ALL), five had acute myeloid leukemia (AML), two had hodgkin lymphoma (HL) and four had non-hodgkin lymphoma (NHL). A total of 127 stool samples were collected among the 39 patients. The average number of days among all samples between chemotherapy start and stool sample collections was 88 days, (median =50 days; min=-5; max=471). Within cancer types ALL patient samples were on average 101.8 days from start of chemotherapy (median=81; min=-4, max=471), AML patient samples were on average 57 days from start of chemotherapy (median=39; min=-5; max=183), HL patient samples were on average 2 days from start of chemotherapy (median=-1; min=-5; max=12), and NHL patient samples were on average 23 days from start of chemotherapy (median=14; min=-1; max=55). Eight patients (ten stool samples) received no antibiotics prior to collection of stools with remaining stool samples collected following some form of antibiotic. Information on the mean days since start of chemotherapy, days of β-lactam use, and vancomycin antibiotics use, for samples in each group are summarized in **Table 1**. Among the 39 patients, 59% experienced one or more gastrointestinal and or blood infection, 26% experienced one or more gastrointestinal infections (predominately *C. difficile*), 51% experienced one or more blood infection, and 18% both gastrointestinal and blood infections. Among the 51% that experienced blood infections 60% of those patients had at least one infection with a bacterium that was identified within the 127 stool samples (including *Streptococcus mitis, Granulicatella adiacens*, *E. coli*, *Enterococcus faecalis, Klebsiella oxytoca, Enterobacter cloacae*, and *Hemophilus influenzae*). Due to timing of sample collection however we could not directly examine if there was a link between the presence of these bacteria in the stool and blood infections.

To avoid potential overrepresentation of one or more patients to a particular group, we used the G-test to evaluate the hypothesis (null) that sampling was approximately uniform among patients within each group. Uniform sampling was rejected in several cases, and backward elimination was employed to remove the patient(s) with the largest number of samples until a uniform distribution could not be rejected in those cases. This resulted in the removal of two patients (12 and 11 samples) in the 1-2 courses of β-lactam, one patient (11 samples) in vancomycin no use, one patient (12 samples) in the 1-2 courses of vancomycin, one patient (12 samples) in the 1-2 courses of AAb, and two patients (10 and 9 samples) in the 3+ courses of AAb (**Supplemental Table S3**). Within the duration groups this resulted in the removal of one patient (12 samples) from the short duration of β-lactam, two patients (10 and 9 samples) from the LM duration of β-lactam, two patient (12 and 9 samples) in short duration of vancomycin, and one patient (10 samples) in the LM duration of vancomycin (**Supplemental Table S3**). Note that there was no treatment-related selection criteria associated with these patients, nor were they outliers with respect to their treatment. They simply were sampled a disproportionally large number of times throughout chemotherapy. As with any opportunistic sampling design, there will be treatment-independent factors that contribute to greater ease of sampling for some patients (Henry, 1990). Here we analyze the sample trimmed datasets (lists of patient IDs and samples used are provided in **Supplemental Table S3**) as a means to reduce the potential for outlier driven bias even though it entails a cost in statistical efficiency.

### 3.2 Taxonomic distribution of sample

Using shotgun metagenome sequence data, 366 taxa were identified in the 127 leukemia and lymphoma samples. This included two Archaea, 348 Bacteria, seven Ascomycota taxa and nine viruses. Mean number of taxa per sample was 42 (min=9, max =115). For species level analyses, only those taxa present in >10% of samples were included, this resulted in 141 taxa (139 Bacteria, 1 Ascomycota, 1 virus) with the mean number of taxa per sample of 38 (min=6, max=89). Among the bacterial taxa, most were members of Firmicutes (83) followed by Bacteroidetes (23), Proteobacteria (16), Actinobacteria (6), and Verrucumicrobia (1) taxa. The distribution of the 141 taxa in the 127 samples are in supplemental material (**Supplementary Figure S2**).

### 3.3 The GIT microbial community changes with repeated courses of antibiotics

**Figure 1:**
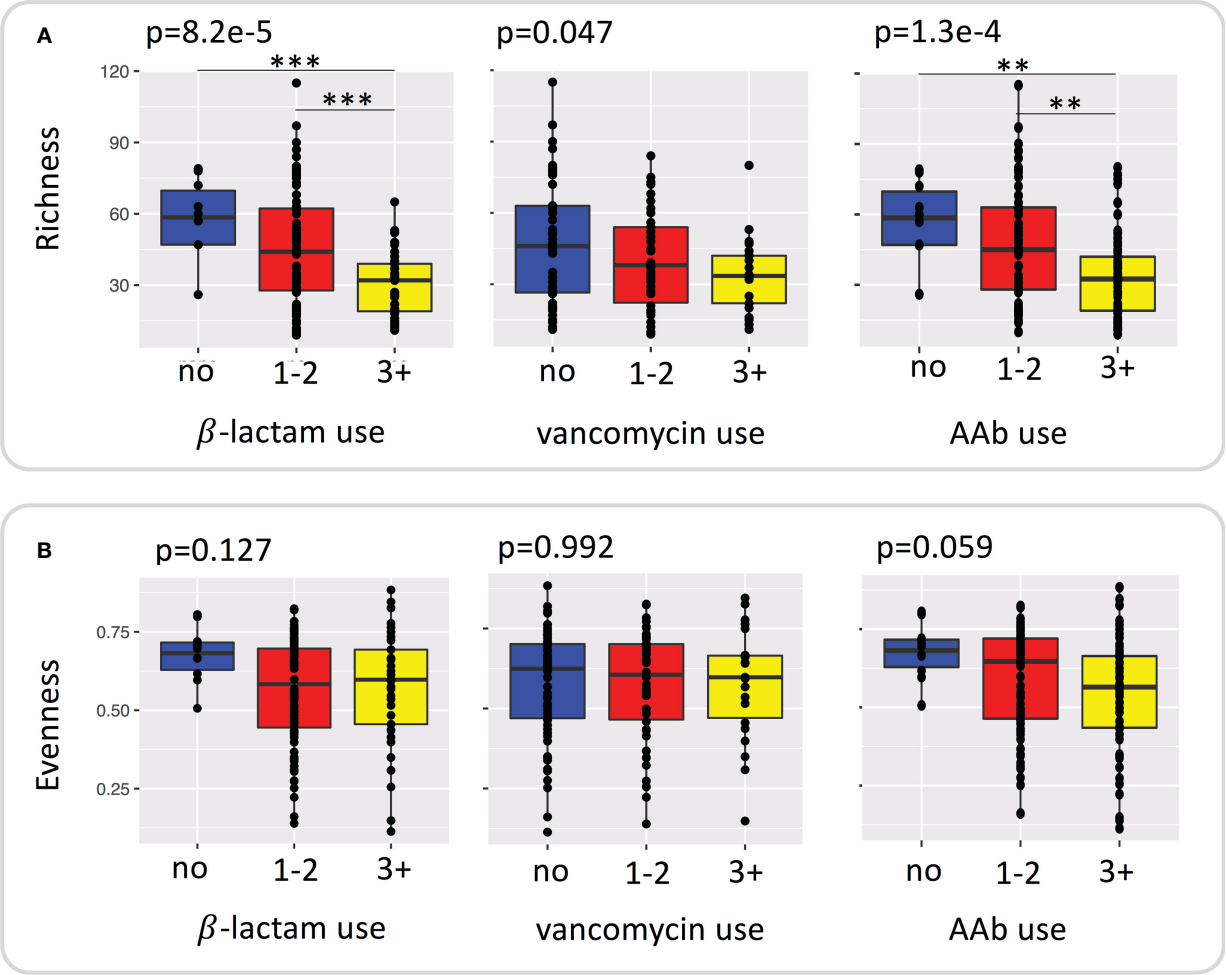
Plot of species **(A)** richness and **(B)** evenness grouped by repeated use of β –lactams, vancomycin, and any antibiotic. Significance was determined using ANOVA with TukeyHSD test for *post hoc* comparisons. ** <0.01, *** < 0.001.

We assessed changes in richness and evenness in samples with repeated courses (no use, 1-2 courses, 3+ courses of β-lactam, vancomycin, AAb) of antibiotics by using ANOVA and Tukey’s *post-hoc* test. Richness (**Figure 1A**) differed significantly with repeated courses of β-lactams (ANOVA p=8.2e-5), vancomycin (ANOVA p=0.047) and AAb (ANOVA p=1.3e-4). **Figure 1A** also shows the individual contrasts with significant differences in richness revealed *via* post-tests, which are consistent with a general decrease in richness with increased courses of β-lactam and AAb. Although the box plots are consistent with a similar relationship for vancomycin, the maximum pairwise difference (no use vs 3+ courses) was only marginally significance (p=0.052) (**Figure 1A**; **Supplementary Table S4**). In contrast to richness, in no cases did evenness differ significantly with number of courses (**Figure 1B**; **Supplementary Table S4**).

We assessed changes in the differential abundance of phyla and species within the microbial community between 1-2 and 3+ courses of β-lactam, vancomycin, and AAb by using ALDEx2. At the phyla level Bacteroidetes decreased significantly with repeated courses (β-lactam, p= 1.8e-4; vancomycin, p=0.012; AAb,p=4.5e-7; **Supplementary Table S5**) while Proteobacteria increased significantly with repeated courses (β-lactams, p=7.3e-7; vancomycin, p=2.0e-4; AAb, p=5.1e-5; **Supplementary Table S5**). Firmicutes increased significantly with repeated courses of β-lactams (p=0.0053) and AAb (p=0.0035) but not for vancomycin (p=0.1079), whereas Fusobacteria increased significantly with repeated courses of vancomycin (p=0.0486**)** and AAb (p=0.005) only (**Supplementary Table S5**).

The species that changed significantly with repeated courses of antibiotics are shown in **Table 2**. The relative abundance of 9 taxa differed significantly with repeated courses of β-lactam antibiotics (p_BH_<0.05). Four species decreased with repeated courses of β-lactam antibiotics (*Eggerthella sp*, and 3 species of Firmicutes). Five species increased in relative abundance with repeated courses of β-lactam antibiotics; these included opportunistic Proteobacteria (*Klebsiella pneumoniae*, and *Alcaligenes* sp.) and Firmicutes (*Enterococcus faecalis, Clostridioides difficile*, and *Streptococcus*). Interestingly, no taxa differed significantly with repeated courses of vancomycin. Examining repeated course of AAb we found that the relative abundance of 18 species differed significantly. Eight species decreased with repeated courses of AAb (*Eggerthella sp*, *Collinsella aerofaciens*, *Alistipes putredinis*, *Bacteroides ovatus* and 4 species in Firmicutes). Ten species increased with repeated courses of AAb (*Rothia mucilaginosa*, along with four species in the phylum Firmicutes and five species in the phylum Proteobacteria). Like β-lactam antibiotics, repeated courses of AAb were associated with an increase in opportunistic pathogens.

**Table 2:** Taxa identified in differential abundance analysis (ALDEx2) as differing significantly with repeated courses (1-2 vs 3+ courses) of β-lactam, vancomycin or AAb.

taxa	wilcoxon p	wilcoxon p_BH_	effect of repeated courses	phylum
β-lactams				
*Eggerthella* sp.	**6.80E-06**	**0.0004**	–	Actinobacteria
*Bifidobacterium longum*	**0.0178**	0.0936	–	Actinobacteria
*Collinsella aerofaciens*	**0.0444**	0.1416	–	Actinobacteria
*Alistipes putredinis*	**0.0115**	0.0580	–	Bacteroidetes
*Clostridium symbiosum*	**0.0005**	**0.0084**	–	Firmicutes
*Faecalibacterium prausnitzii*	**0.0015**	**0.0195**	–	Firmicutes
*Oscillibacter* sp.	**0.0026**	**0.0252**	–	Firmicutes
*Ruminococcus obeum*	**0.0192**	0.0748	–	Firmicutes
*Clostridium bolteae*	**0.0254**	0.1114	–	Firmicutes
*Enterococcus faecalis*	**0.0003**	**0.0047**	+	Firmicutes
*Clostridioides difficile*	**0.0045**	**0.0313**	+	Firmicutes
*Streptococcus mitis oralis pneumoniae*	**0.0068**	**0.0467**	+	Firmicutes
*Streptococcus peroris*	**0.0148**	0.0660	+	Firmicutes
*Granulicatella adiacens*	**0.0174**	0.0746	+	Firmicutes
*Enterococcus faecium*	**0.0251**	0.0921	+	Firmicutes
*Granulicatella* sp.	**0.0240**	0.0986	+	Firmicutes
*Streptococcus parasanguinis*	**0.0453**	0.1589	+	Firmicutes
*Klebsiella pneumoniae*	**0.0008**	**0.0097**	+	Proteobacteria
*Alcaligenes* sp.	**0.0079**	**0.0430**	+	Proteobacteria
*Brevundimonas diminuta*	**0.0147**	0.0605	+	Proteobacteria
*Bordetella* sp.	**0.0141**	0.0615	+	Proteobacteria
*Escherichia coli*	**0.0169**	0.0786	+	Proteobacteria
*Agrobacterium tumefaciens*	**0.0222**	0.0851	+	Proteobacteria
*Agrobacterium* sp.	**0.0216**	0.0871	+	Proteobacteria
*Achromobacter piechaudii*	**0.0293**	0.1084	+	Proteobacteria
vancomycin				
*Eggerthella* sp.	**0.0371**	0.3778	–	Actinobacteria
*Enterococcus faecalis*	**0.0185**	0.3222	+	Firmicutes
*Clostridioides difficile*	**0.0244**	0.3612	+	Firmicutes
*Streptococcus parasanguinis*	**0.0304**	0.4243	+	Firmicutes
AAb				
*Eggerthella* sp.	**0.0004**	**0.0054**	–	Actinobacteria
*Collinsella aerofaciens*	**0.0097**	**0.0443**	–	Actinobacteria
*Bifidobacterium longum*	**0.0182**	0.0771	–	Actinobacteria
*Alistipes putredinis*	**0.0001**	**0.0021**	–	Bacteroidetes
*Bacteroides ovatus*	**0.0004**	**0.0055**	–	Bacteroidetes
*Bacteroides caccae*	**0.0229**	0.0764	–	Bacteroidetes
*Alistipes onderdonkii*	**0.0275**	0.0832	–	Bacteroidetes
*Parabacteroides merdae*	**0.0329**	0.1028	–	Bacteroidetes
*Faecalibacterium prausnitzii*	**0.0000**	**0.0008**	–	Firmicutes
*Oscillibacter* sp.	**0.0016**	**0.0139**	–	Firmicutes
*Clostridium symbiosum*	**0.0033**	**0.0222**	–	Firmicutes
*Ruminococcus obeum*	**0.0037**	**0.0226**	–	Firmicutes
*Clostridium bolteae*	**0.0391**	0.1201	–	Firmicutes
*Rothia mucilaginosa*	**0.0031**	**0.0213**	+	Actinobacteria
*Bifidobacterium animalis*	**0.0424**	0.1034	+	Actinobacteria
*Enterococcus faecalis*	**0.0008**	**0.0078**	+	Firmicutes
*Clostridioides difficile*	**0.0022**	**0.0150**	+	Firmicutes
*Granulicatella adiacens*	**0.0047**	**0.0224**	+	Firmicutes
*Granulicatella* sp.	**0.0118**	**0.0447**	+	Firmicutes
*Streptococcus peroris*	**0.0150**	0.0524	+	Firmicutes
*Streptococcus mitis oralis pneumoniae*	**0.0168**	0.0659	+	Firmicutes
*Enterococcus faecium*	**0.0246**	0.0748	+	Firmicutes
*Ruminococcus gnavus*	**0.0230**	0.0850	+	Firmicutes
*Blautia producta*	**0.0497**	0.1177	+	Firmicutes
*Klebsiella pneumoniae*	**0.0019**	**0.0125**	+	Proteobacteria
*Alcaligenes* sp.	**0.0075**	**0.0306**	+	Proteobacteria
*Brevundimonas diminuta*	**0.0070**	**0.0321**	+	Proteobacteria
*Agrobacterium tumefaciens*	**0.0117**	**0.0421**	+	Proteobacteria
*Agrobacterium* sp.	**0.0114**	**0.0451**	+	Proteobacteria
*Achromobacter piechaudii*	**0.0191**	0.0608	+	Proteobacteria
*Bordetella* sp.	**0.0274**	0.0716	+	Proteobacteria
*Escherichia coli*	**0.0243**	0.0821	+	Proteobacteria

Bolded values indicate values <0.05. Analyses were performed on the reduced patient dataset (see **Supplemental Table S3** for breakdown of samples and patients).

The overall trend of taxonomic change with repeated course of antibiotics was a decrease in relative abundance in taxa important for gut health and integrity (*Clostridium symbiosum*, Oscillibacter, *Faecalibacterium prausnitzii, Ruminococcus obeum, Eggerthella sp*) including those involved in bile acid and short chain fatty acid production. In addition, many of the taxa that increased in abundance with repeated courses (*Granulicatella adiacens*, *Enterococcus faecalis, Streptococcus, Clostridioides difficile* and *Klebsiella pneumonia*) have been implicated in infections in immunocompromised patients.

### 3.4 The GIT microbial community changes with duration of antibiotics

To examine changes in richness and evenness in samples with increased duration of antibiotics (no use, short, LM of β-lactams and vancomycin) we used ANOVA and Tukey’s test. A significant effect of duration on species richness was only observed for β-lactams, where richness decreased with increasing duration (**Supplementary Table S4**). There was no relationship between evenness and the duration of either β-lactams or vancomycin treatment.

We investigated the impact of short versus LM duration of antibiotic treatment (β-lactams and vancomycin) on the relative abundance of individual taxa within the microbial community by using ALDEx2. At the phylum level, longer duration of β-lactam was associated with a significant decrease in relative abundance of Bacteroidetes (p=5.2e-4), and increase in Proteobacteria, and Fusobacteria (p=5.7e-4; and p=0.0067; respectively) (**Supplementary Table S5**). With increased duration of vancomycin only Proteobacteria was significantly changed, showing an increase in relative abundance (p=0.0191; **Supplementary Table S5**). At the species level, we identified no lineages that differed significantly between short and LM duration of β-lactams or vancomycin when we corrected p-values for multiple tests (**Table 3**).

**Table 3:** Taxa identified in differential abundance analysis (ALDEx2) as differing significantly with increased duration (short vs LM) of β-lactams or vancomycin.

taxa	wilcoxon p	wilcoxon p_BH_	effect of increased duration	phylum
β-lactams				
*Eggerthella* sp.	**0.0100**	0.0927	–	Actinobacteria
*Collinsella aerofaciens*	**0.0189**	0.1117	–	Actinobacteria
*Bifidobacterium longum*	**0.0204**	0.1298	–	Actinobacteria
*Parabacteroides merdae*	**0.0200**	0.1128	–	Bacteroidetes
*Alistipes putredinis*	**0.0383**	0.1545	–	Bacteroidetes
*Bacteroides ovatus*	**0.0478**	0.1859	–	Bacteroidetes
*Faecalibacterium prausnitzii*	**0.0204**	0.1324	–	Fimicutes
*Rothia mucilaginosa*	**0.0172**	0.1079	+	Actinobacteria
*Streptococcus mitis oralis pneumoniae*	**0.0104**	0.0955	+	Fimicutes
*Streptococcus peroris*	**0.0196**	0.1146	+	Fimicutes
*Streptococcus australis*	**0.0408**	0.1546	+	Fimicutes
*Streptococcus sanguinis*	**0.0470**	0.1678	+	Fimicutes
*Enterobacter cloacae*	**0.0086**	0.0756	+	Proteobacteria
*Agrobacterium tumefaciens*	**0.0190**	0.1156	+	Proteobacteria
*Brevundimonas diminuta*	**0.0216**	0.1187	+	Proteobacteria
*Agrobacterium* sp.	**0.0247**	0.1328	+	Proteobacteria
*Achromobacter piechaudii*	**0.0285**	0.1393	+	Proteobacteria
*Escherichia* sp.	**0.0291**	0.1543	+	Proteobacteria
*Alcaligenes* sp.	**0.0338**	0.1553	+	Proteobacteria
vancomycin				
*Flavonifractor plautii*	0.0328	0.6548	+	Fimicutes
*Escherichia* sp.	0.0140	0.6389	+	Proteobacteria
*Enterobacter cloacae*	0.0324	0.6443	+	Proteobacteria

Bolded values indicate values <0.05. Analyses were performed on the reduced patient dataset (see **Supplemental Table S3** for breakdown of samples and patients per group).

### 3.5 A wide variety of resistance genes were identified in pediatric leukemia and lymphoma patients

We identified 264 ARGs spanning 24 antibiotic classes present in at least 1 of the 127 leukemia and lymphoma patient samples (**Supplementary Table S6**). We found that 254 of these genes occurred in ≥2 samples and 200 of these ARGs occurred in ≥5% of the 127 samples (>6 samples) (**Supplementary Table S6**). Analyses were performed using the 200 genes (23 antibiotic classes) identified in ≥5% of samples (**Supplementary Tables S6 and S7**). Most ARGs (69) were connected to resistance in multiple drugs (coded as multidrug), followed by ARGs belonging to the following antibiotic resistance classes: glycopeptide (19), β-lactam (18), tetracycline (18), aminoglycoside (15) and peptide (13) antibiotics. The remaining 49 ARGs were spread across 17 antibiotic classes (**Supplementary Table S7**). The high occurrence of ARGs in glycopeptide and peptide antibiotic classes, which contain the vancomycin resistance genes, and the β-lactam antibiotic class, which contains resistance genes to β-lactam antibiotics was not surprising, as vancomycin (a glycopeptide antibiotic) and piperacillin-tazobactam (a β-lactam antibiotic) were the most used drugs in our patients (46 and 82% of patients respectively). Based on this, we further investigated the effect of the repeated courses, or duration, of *β-lactam and vancomycin* antibiotics as well as combined antibiotic use on the 119 resistance genes from the following four antibiotic classes: β-lactam (18 genes), glycopeptide (19 genes), peptide (13 genes), and multidrug (69 genes). These classes were specifically selected as the β-lactam antibiotic class and multidrug antibiotic class potentially contains genes for resistance to β-lactam antibiotics, and the glycopeptide antibiotic class, peptide antibiotic class and multidrug antibiotic class potentially contains genes for resistance to vancomycin.

### 3.6 The abundance of resistance genes within the GIT microbiome changes with increased courses of antibiotics

#### 3.6.1 β-lactam ARGs

Examining significant and candidate genes with repeated courses measured either as course groups or counts of courses identified 9 β-lactam ARGs, with three genes (ACT-2, ampH, ompK37) increased with increased courses (**Table 4**). A total of 8 β-lactam resistance genes differed with repeated course groups (**Supplementary Table S8**); Six genes (cblA-1, CBP-1, cepA, cfxA2, cfxA4, cfxA6) decreased in relative abundance and two (ampH, ompK37) increased (**Supplementary Table S8**). Assessing repeated use as a measure of number of courses of an antibiotic also identified 8 β-lactam resistance genes with number of vancomycin or AAb courses, six were the same as those identified with repeated course, with ACT-2 replacing cblA-1, resulting in three genes (ACT-2, ampH, ompK37) identified with number of courses of AAb (**Supplementary Table S9**).

**Table 4:** Combined list of significant and candidate (correcting for confounders; p_BH_<0.1; p_BH_<0.05 in bold) ARGs from the ß-lactam, glycopeptide, peptide and multidrug antibiotic classes for different mean abundances with repeated courses (no use, 1-2 courses, 3+ courses), and/or counts of courses of ß-lactam, vancomycin or AAb.

	*repeated courses of ß-lactams*		*repeated courses of vancomycin*		*repeated courses of Aab*	
	increased	decreased	increased	decreased	increased	decreased
***ß-lactam genes***	ampH, ompK37	cblA-1, CBP-1, cepA,	**ACT-2**	cfxA2	ACT-2, ampH, ompK37	**cblA-1, CBP-1**, **cepA**, **cfxA2**, **cfxA4**, **cfxA6**
**Glycopeptide genes**		**vanRD**, **vanUG**, **vanVB**, **vanYB,** vanG, vanWG,		**vanVB, vanYB**		**vanUG**, **vanVB**, **vanRD, vanYB**
**Peptide genes**	arnA, eptA, eptB, pmrF, yojI				**arnT, pmrF, yojI,** arnA, bacA, eptB, liaS, rosB, ugd	
**Multidrug genes**	**acrF**, acrA, acrR, baeR, cpxA, kpnF, lsaA, mdfA, sdiA, soxR, soxS, tolC	**ermF**, lsaB, tetX	**acrB**, **ramR**		**acrR**, **adeF**, **baeS**, **H-NS, kpnG, lsaA, marA, oqxA**, **oqxB, ramA**, **soxR, soxS,** acrA, acrB, acrE, acrF, adeF, cpxA, kpnE, kpnF, lsaC mdfA, mdtM, ramR, rsmA, sdiA, tolC	**ermF**, **msrE**, **tetX**

Analyses were performed on 104 samples from 37 patients for ß-lactam and vancomycin and 96 samples from 38 patients for AAb (see **Supplemental Table S3** for breakdown of samples and patients per group). Significance was determined using linear models in LIMMA with cancer diagnosis and days from start of chemotherapy treated as confounders, and significance was corrected for multiple tests using BH.

#### 3.6.2 Glycopeptide ARGs

Examining significant and candidate genes with repeated courses measured either as course groups or counts of courses identified 6 glycopeptide ARGs, all decreased with increased courses (**Table 4**). A total of six glycopeptide resistance genes differed with repeated course groups (**Table 4**; **Supplementary Table S8**); five with repeated β-lactam courses, 1 with repeated vancomycin courses, and 3 with repeated AAb courses. In all cases, the genes decreased in relative abundance with repeated course (**Table 4**; **Supplementary Table S8**). Assessing repeated use as a measure of number of courses of an antibiotic identified five glycopeptide resistance genes that differed (**Table 4**; **Supplementary Table S9**), 5 with repeated β-lactam courses, 2 with repeated vancomycin courses, and 4 with repeated AAb courses.

#### 3.6.3 Peptide ARGs

Examining significant and candidate genes with repeated courses measured either as course groups or counts of courses identified 10 peptide ARGs, with all increased with increased courses (**Table 4**). A total of six peptide resistance genes differed with repeated course groups (**Supplementary Table S8**); 5 with repeated β-lactam and 2 with repeated AAb courses, while none were significant with repeated vancomycin courses (**Supplementary Table S8**). Assessing repeated use as numbers of courses of an antibiotic, identified 8 peptide resistance genes with course of AAb, 4 were the same as those identified with repeated course, however, liaS, rosB, arnT, and bacA were also identified (**Supplementary Table S9**).

#### 3.6.4 Multidrug ARGs

Examining significant and candidate genes with repeated courses measured either as course groups or counts of courses identified 31 multidrug ARGs with most (27) increased with increased courses (**Table 4**). A total of 20 multidrug resistance genes differed with repeated course groups (**Supplementary Table S8**); 14 genes were identified for repeated β-lactam courses, 8 with repeated AAb courses and none with repeated vancomycin courses. Most genes increased with repeated course (β-lactams 12/14; AAb 6/8; **Table 4**). Only two genes were identified in both repeated courses of β-lactam and AAb, and both (ermF, and tetX) decreased with repeated courses (**Table 4**; **Supplementary Table S8**). Assessing repeated use as numbers of courses of an antibiotic, identified 27 multidrug genes (one with β-lactam courses; two with vancomycin courses; and 26 with AAb courses), 16 of which were also identified with repeated course (**Supplementary Table S9**).

### 3.7 The abundance of resistance genes changes within the GIT microbiome with increased duration or days of antibiotics

#### 3.7.1 β-lactam ARGs

Examining significant and candidate genes with increased duration measured either as duration groups or days of antibiotics identified 14 β-lactam ARGs, with 10 genes increased with increased duration and or days (**Table 5**). A total of 5 β-lactam resistance genes differed with increased duration of β-lactams or vancomycin (**Supplementary Table S10**). Three of these genes (PBP1a, PBP2x, PBP2b) increased in relative abundance with increased duration of β-lactams and one (ACT-2) increased in relative abundance with increased duration of vancomycin (**Supplementary Table S10**). Assessing increased duration of antibiotic use as days of antibiotic given, we identified 14 genes (12 with days of β-lactam; 9 with days of vancomycin), including all 5 of those identified with increased duration. Six (ACT-2, ampC, ampC1, ampH, ompA, ompK37) of the genes identified increased in abundance with increasing days of antibiotics (**Supplementary Table S11**).

**Table 5:** Combined list of significant (correcting for confounders; p<0.05; p_BH_<0.1; p_BH_<0.05 bolded) ARGs from the ß-lactam, glycopeptide, peptide and multidrug antibiotic classes with increased duration (no use, short duration, LM duration) and/or days of ß-lactam or vancomycin.

	*increased duration/days of ß-lactams*		*increased duration/days of vancomycin*	
	increased genes	decreased genes	increased genes	decreased genes
***ß-lactam genes***	**ACT-2**, **PBP1a, PBP2x,** ampH, LEN32, ompA, ompK37, PBP2b	cblA-1, cepA, cfxA2, cfxA6	**ACT-2**, **ampC**, **ampH**, **ompA**, **ompK37,** ampC1,	**cfxA2**, **cfxA6,** cblA-1
**Glycopeptide genes**		vanRD, vanYB		**vanRD, vanUG, vanVB**, **vanYB,** vanG, vanTG, vanWG
**Peptide genes**	**arnT**, **bacA**, **pmrF**, **yojI**, arnA, eptB, rosB,		**arnA**, **arnT**, **bacA**, **eptB**, **pmrF**, **rosB**, **ugd**,**yojI**,	
**Multidrug genes**	**acrB**, **adeF**, **baeR**, **baeS**, **kpnG**, **marA**, **mdfA**, **oqxA**, **ramA**, **ramR**, **rsmA, sdiA**, **soxS**, acrA, acrE, acrR, cpxA, CRP, H-NS, kpnE, kpnF, lsaC, mdtM, mel, soxR, tolC	**msrE,** mtrA	**acrA**, **acrB**, **acrE**, **acrR**, **acrS**, **adeF**, **baeR**, **baeS**, **cpxA**, **CRP**, **evgA**, **evgS**, **gadW**, **gadX**, **H-NS, kpnE**, **kpnF**, **kpnG**, **lptD**, **marA**, **mdfA**, **mdtE**, **mdtF**, **mdtO**, **mdtP**, **oqxA**, **ramA**, **ramR**, **rsmA**, **sdiA**, **soxR**, **soxS**, **tolC**	**efrB, msrE**

Analyses were performed on 96 samples from 38 patients for ß-lactam and 85 samples from 37 patients for vancomycin (see **Supplemental Tables S3** for breakdown of samples and patients per group). Significance was determined using linear models in LIMMA with cancer diagnosis and days from start of chemotherapy treated as confounders, and significance was corrected for multiple tests using BH.

#### 3.7.2 Glycopeptide ARGs

Examining significant and candidate genes with increased duration measured either as duration groups or days of antibiotics identified seven glycopeptide ARGs, all decreased with increased duration and or days (**Table 5**). A total of three glycopeptide resistance genes differed with increased duration of vancomycin courses, while no genes were identified with duration of β-lactams. All genes identified decreased in relative abundance with increased duration of vancomycin (**Supplementary Table S10**). Assessing increased duration of antibiotic use as days of antibiotic given, we identified seven genes (2 with days of β-lactam; 7 with days of vancomycin), with all three of the genes identified with increased duration (vanG, vanUG, vanYB) also identified with days given. All genes identified decreased in relative abundance as antibiotic days increased (**Supplementary Table S11**).

#### 3.7.3 Peptide ARGs

Examining significant and candidate genes with increased duration measured either as duration groups or days of antibiotics identified eight peptide ARGs, with all genes increased with increased duration and or days (**Table 5**). Only one peptide resistance genes (ugd) significantly increased in relative abundance with increased duration groups of vancomycin (**Table 5**; **Supplementary Table S10**). Assessing increased duration of antibiotic use as days of antibiotic given, we identified seven genes with both days of β-lactam and days of vancomycin, all with increased relative abundance as antibiotic days increased (**Table 5**, **Supplementary Table S11**).

#### 3.7.4 Multidrug ARGs

Examining significant and candidate genes with increased duration measured either as duration groups or days of antibiotics identified 39 multidrug ARGs, with 36 genes increased with increased duration and or days (**Table 5**). Two multidrug resistance genes (acrB, ramR) increased with increased duration of vancomycin courses (**Supplementary Table S10**). Assessing increased duration of antibiotic use as days of antibiotic given, we identified 39 genes (26 with days of β-lactam; 35 with days of vancomycin), with both of the genes identified with increased duration also identified. Most genes increased in relative abundance as antibiotic days increased (24/26 β-lactam; 33/35 vancomycin; **Supplementary Table S11**).

### 3.8 Correlation between relative abundance of taxa and ARGs across all samples

**Figure 2:**
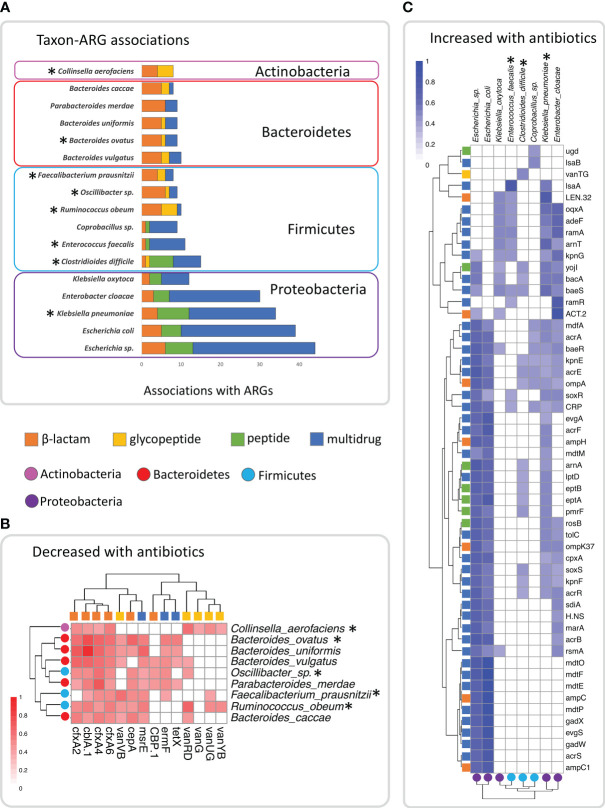
Plots showing the significant (p_BH_<0.05) spearman correlations between species and the ARGs identified in this study in the 127 samples, showing the top 18 taxa with the most significant associations. **(A)** Plot of number of significant associations with ARGs in the top 18 taxa, indicating the number within each of the four antibiotic classes (β –lactam, glycopeptide, peptide and multidrug). **(B)** Heatmap of Spearman correlation coefficients of ARGs and taxa among the top 18 that clustered together and decreased in abundance with repeated courses or increased duration of antibiotics. **(C)** Heatmap of Spearman correlation coefficients of ARGs and taxa among the top 18, that clustered together and were found to increase in abundance with repeated courses or increased duration of antibiotics. Taxa indicated with asterisks were significant based on ALDEx2 analysis.

Because both genes and taxa were impacted by antibiotic use, and it is not possible to directly infer which taxa were contributing ARGs (due to the lack of genomic context, and the potential for LGT between genomes), we used Spearman correlation analysis to investigate associations between the abundance of the 141 species and the 78 ARGs we identified as associated with antibiotic use (**Tables 4**, **5**). We found significant (p_BH_<0.05) associations in 512 of the 10,998 tested comparisons between taxa and ARGs (**Supplementary Table S12**). We found that over half of these (274) were associated with just 18 taxa across four phyla (**Figure 2A**). The four taxa with the most correlations (*Klebsiella pneumonia, Escherichia* sp.*, E. coli*, and *Enterobacter cloacae*) were all from the phylum Proteobacteria (**Figure 2A**). All five species of Proteobacteria we identified were predominately associated with multidrug and peptide genes, and all were opportunistic pathogens. Species in the phyla Bacteroidetes, and Firmicutes were predominately associated with β-lactam and multidrug ARGs in addition to a few glycopeptide genes (**Figure 2A**). The exception to this was the Fimicutes, *Clostridioides difficile, Enterococcus faecalis*, and *Coprobacillus* sp., which had associations with peptide genes. *Clostridioides difficile* in particular had counts of associations with genes in antibiotic classes which were much more similar to lineages in Proteobacteria than to Firmicutes (**Figure 2A**).

Analysis of the significant gene-taxon correlation coefficients showed two distinct clusters of gene-taxon pairs (**Figures 2B, C**). The taxa within one cluster (**Figure 2B**) contained lineages from Actinobacteria, Bacteroidetes and Firmicutes, with most of these taxa having been identified in our previous differential abundance analysis as decreased with repeated courses and duration of antibiotics (**Tables 2**, **3**). In addition, the ARGs within this cluster were those β-lactam, glycopeptide, and multidrug genes that we previously identified as decreased with repeated courses and duration of antibiotics (**Tables 4**, **5**). These results suggest that there may exist a unique subset of taxa that decrease jointly with certain ARGs (likely because they encode those ARGs in their genomes) either in response to the sensitivity of those taxa to repeated courses and duration of antibiotics or to the broader ecological changes within the GIT microbiome caused by those antibiotics.

The second cluster (**Figure 2C**) contained lineages from Proteobacteria and *C. difficile*, *Enterococcus faecalis*, and *Coprobacillus* sp., three Firmicutes taxa, two of which increased with repeated courses of antibiotics (**Table 2**). In addition, taxa in this cluster included associations with peptide genes (**Figure 2A**), and a different set of β-lactam, and multidrug genes than in cluster one (**Figure 2C**). Most of the ARGs in this cluster were genes that we previously identified as increased with repeated courses and duration of antibiotics (**Tables 4**, **5**) with the only exceptions being vanTG and lsaB. These results suggest that there may exist a different subset of taxa that increase jointly with certain ARGs (likely because they encode them) in response to the ecological changes within the GIT microbiome caused by repeated courses and duration of antibiotics.

## 4 Discussion

We used paired-end shotgun metagenome sequencing to evaluate the abundance of ARGs in stool samples collected from pediatric patients undergoing treatment for leukemia and lymphoma. Use of antibiotics reduces mortality, but the risk of infection in leukemia and lymphoma patients based on reduced immune status and/or neutrophil count is not known, and extended use of empiric antibiotics may negatively impact patient outcomes by increasing resistant organisms. The overall effect on antibiotic resistance is not well understood in children who receive multiple courses and long duration antibiotics during cancer treatment. Unlike previous studies that compared short sequence fragment to the CARD database of ARGs (Margolis et al., 2021), we used a different approach based on complete or nearly complete genes identified from contiguous sequences from a set of diverse pediatric oncology patients. Our new pediatric oncology ARG database was then used to map sequence reads from stool samples taken from leukemia and lymphoma patients subjected to varying antibiotic use and duration to see how ARGs varied with antibiotics. Mapping larger complete or nearly complete gene fragments allows for more accurate assignment to the CARD database. However, the increased accuracy of this approach does entail lower power to the extent that short reads not represented in the pediatric oncology ARG database will not be identified. While our results represent a high-quality baseline, there is the potential that even more ARGs could be present and identifiable in the future with the mapping of all reads to the CARD database. Furthermore, while this approach is based on complete or nearly complete genes identified from contiguous sequences it does not address allelic (mutant) variation or SNPs that result in antibiotic resistance but does narrow the pool of genes for future deep sequencing to examine these variations.

Samples in this study were heterogeneous in the number and duration of antibiotics received prior to collection. This permitted us to examine the effect of repeated courses and duration of commonly prescribed antibiotics for febrile neutropenia (β -lactams and vancomycin) on the resistome in the gastrointestinal tract. We examined the abundance of specific ARGs and taxa related to β-lactam antibiotics (β-lactam genes), vancomycin (glycopeptide and peptide antibiotic genes) and multi-drug resistance. We found that there were sets of taxa and genes that increased or decreased in relative abundance after repeated courses of β-lactams and vancomycin and with longer duration of these antibiotics. Many of the significantly changing taxa and identified genes were positively correlated with each other, increasing and decreasing in abundance together.

Broad-spectrum β-lactam antibiotics are meant to treat both Gram-positive and Gram-negative bacteria. While we found that the overall richness (number of species) declined with repeated β-lactam antibiotic courses and increased duration, which was reflected in the decreased abundance of Bacteroidetes, we found the relative abundance of Proteobacteria and Firmicutes phyla increased. Closer examination of Firmicutes taxa identified opportunistic taxa like *Granulicatella, Streptococcus*, *Clostridioides difficile* and *Enterococcus faecalis* increased in abundance with repeated courses of β-lactams. Conversely, other “good” Firmicutes like *Faecalibacterium prausnitzii, Oscillibacter*, *Clostridium symbiosum*, and *Ruminococcus obeum*, that help keep pathogenic and opportunistic pathogens in check (Pultz et al., 2005; Taur et al., 2012; Keith and Pamer, 2019) through competition or host immune education (Hill and Artis, 2010; Zhao and Elson, 2018; Cheng et al., 2019) were decreased in abundance with repeated courses of β-lactams. Since these taxa are associated with general host health (Machiels et al., 2014; Morrison and Preston, 2016; Parada Venegas et al., 2019; Deleu et al., 2021), our results suggest more systemic health effects may be an outcome of repeated use of β-lactams. Among the Proteobacteria, we identified opportunistic pathogens increased in relative abundance, including *Klebsiella pneumoniae*, *Alcaligenes* sp., and *Escherichia coli*. These bacteria, while normally present throughout the GIT, typically occur at a very low relative abundance (Hollister et al., 2014; Keith and Pamer, 2019). In addition to our study, Keith and Pamer (2019) found increased prevalence of resistant *E. coli* and *K. pneumoniae* in clinical care settings with increased antibiotic use, and Taur et al. (2012) observed increased abundance of opportunistic pathogens, including *Enterococcus*, *Streptococcus*, and Proteobacteria taxa in patients undergoing antibiotic treatment following hematopoietic stem cell transplantation, which increased the risk of bacteremia.

Vancomycin is more narrowly focused than broad-spectrum β-lactams in its target and is routinely used to treat Gram-positive bacteria. While richness declined with vancomycin use, it was not significant for either repeated courses or duration. We postulate that this was a result of the overlap of samples (36/46) that received no vancomycin but had received at least one course of a β-lactam antibiotics. We showed that use of β-lactam in this population changes the relative abundance of taxa in general, and Firmicutes taxa in particular; hence, its use is likely causing a significant change in the microbiome community that makes it more difficult to detect taxonomic shifts that result from vancomycin use.

Resistance to β-lactams can be achieved by inactivation, or modification of the antibiotic *via* β-lactam gene (*e.g.*, cepA, cfxA, cfiA, PBP) products and/or through overexpression of multidrug efflux pump genes (Pumbwe et al., 2006; García et al., 2008). If repeated courses of β-lactams are impacting the composition of the bacterial resistome community we would anticipate increased resistance genes in samples collected after repeated courses or duration. While we did find β-lactam genes in the resistome, most were decreased with β-lactam use. This is likely because these genes, which initially emerged to protect bacteria from naturally occurring β-lactams (Bush, 2018), are not conferring resistance to these taxa and their decline is a result of decreased relative abundance of taxa containing these genes. In fact, many of the taxa that were correlated with these genes were also decreased with β-lactam use. Similarly, other researchers have found bacteria carrying β-lactam resistance genes (*e.g*., cfxA) were sensitive to β-lactam antibiotics (Wilke et al., 2005; Xie et al., 2014; Binta and Patel, 2016). While this study identified genes matching resistance genes it does not address mutant variation or SNPs in these genes that can be responsible for antibiotic resistance. In addition, some β-lactam genes require induction to result in resistance, so gene presence alone does not contribute to resistance (Binta and Patel, 2016); however, their presence in the gut community does require monitoring.

We did find, however, that multidrug resistance genes were increased with repeated courses and duration of β-lactams. Note that among the 45 identified significant or candidate multidrug genes, over half (53%) included a β-lactam class as a target. In addition, most of the increased multidrug genes (92%) were antibiotic efflux genes, and many of these efflux genes were resistance-nodulation-division (RND) efflux pumps. Identified efflux genes included multiple genes creating proteins complexes: acrA, acrB and tolC (AcrAB-TolC efflux pump); kpnE and kpnF (KpnEF efflux pump); acrE and acrF (AcrEF efflux pump). In addition to the efflux gene, we also identified multidrug genes that regulated these efflux pump genes, including: acrR, acrS, marA, H-NS, cpxA, sdiA, soxS, and ramA. The identified multidrug efflux-related genes were predominately correlated with *Escherichia sp*, *E. coli*, *Enterobacter cloacae*, *Klebsiella pneumonia* and *Clostridioides difficile*, taxa that generally increased with antibiotic use. These efflux related genes, and associated taxa, have been shown to be involved in resistance in clinical settings in *Klebsiella pneumonia*, *E. coli*, and *Enterobacter cloacae* (Pérez et al., 2012; Bialek-Davenet et al., 2015; Davin-Regli and Pagès, 2015; Davin-Regli et al., 2019; Maurya et al., 2019). Finally, the close similarity between *C. difficile* gene association and the above opportunistic bacteria suggests potential lateral gene transfer as a mechanism for this similarity, as many of the efflux genes are not normally present in Gram-positive bacteria (Nikaido, 2011; Lamut et al., 2019; Ebbensgaard et al., 2020). It is of particular concern that increases in pathogens are associated with increases in multidrug resistant genes, as this suggests that these taxa are likely to be resistant to other antibiotics and they could put pediatric oncology patients at higher risk of serious untreatable infections.

Efflux pumps have been shown to be important in pathogenicity, along with bacterial metabolism, and physiology (Piddock, 2006). They play a role in quorum sensing, adherence, invasion and colonization of host cells, biofilm formation, and survival in the presence of toxins, heavy metals, and antimicrobials (Evans et al., 1998; Piddock, 2006; Anes et al., 2015; Alav et al., 2018). Efflux pumps enable bacteria to survive in part by regulation of stress through stress responses and controlling their environment (Webber and Piddock, 2003; Blanco et al., 2016; Du et al., 2018). Bacterial stress response encourages adaptation for survival (Tan et al., 2022) and in pathogenic bacteria promotes virulence in stressful environments (Flores-Kim and Darwin, 2014). Genes that regulate bacterial stress response (e.g., marA, soxS, and sdiA) also regulate efflux pump (e.g., acrA, acrB, tolC that form AcrAB-TolC efflux pump) multidrug resistance genes (Giuliodori et al., 2007). This suggests that activation of stress response as the result of environmental stresses could also lead to antibiotic resistance (Giuliodori et al., 2007; Fang et al., 2016). Gram-negative bacteria that are found to be more resistant to stress also frequently contain virulence factors, and lead to bloodstream and gastrointestinal infections (Morris and Cerceo, 2020). In this study we found that 59% of the patients experienced at least one GI and or blood infection, and among those that experienced blood infections 60% of patients had at least one infection with a bacterium identified within the 127 stool samples including opportunistic pathogens (*E. coli*, *Enterococcus faecalis, Klebsiella oxytoca, and Enterobacter cloacae*) with increased relative abundance and associated with increased multidrug genes related to efflux pumps.

Given the strong associations between these opportunistic pathogens and multidrug genes related to efflux pumps, we suggest these genes may be important in allowing these taxa to persist or increase in abundance with repeated courses and duration of antibiotics. While the most direct explanation involves their ability to remove antibiotics, other mechanisms that confer enhanced ability to handle environmental stress, or other aspects of the treatment environment, could contribute to their ability to flourish within the gut microbiome during treatment.

Finally, we note increases in some ARGs from one antibiotic class were associated with exposure to another antibiotic class. Although this finding is significant in terms of monitoring and predicting the prevalence of ARGs in a clinical setting, the phenomenon should not be taken as evidence of a causal functional role for such genes within the treatment environment. Antibiotic exposure results in ecological changes that shift community composition of the human microbiome (Dethlefsen et al., 2008; Dethlefsen and Relman, 2011; Iizumi et al., 2017), and these new community states will plausibly involve high prevalence of some lineages that carry genes from another antibiotic class. Given that bacterial LGT is frequent among many lineages (and gene composition will be variable), increases in the relative frequency of some classes of ARGs are expected and yet could be difficult to predict. Collectively, these findings raise the possibility that ongoing monitoring of the distribution of ARGs over clinically relevant timeframes and locations may be warranted.

Chemotherapy can alter the microbiome resulting in dysbiosis which can lead to diarrhea and further depletes the healthy gut microbes (Montassier et al., 2015). To attempt to account for this we used cancer diagnosis and days from start of chemotherapy as confounders in our model. The use of convenience samples rather than specified timepoints was a limitation in this study. Although convenience sampling is cheap and efficient to implement (Henry, 1990), in our case it led to some patients being sampled a disproportionally large number of times throughout chemotherapy. We removed them from subsequent analyses, with a potential cost to statistical efficiency, because we could not determine if these represented a source of bias. To better understand the role of chemotherapy in microbial dysbiosis, and to further evaluate the changes in ARGs relating to antibiotic use, a more formal sampling design including explicit time-point dependent sampling may be necessary. In addition, all children received trimethoprim-sulfamethoxazole prophylactically throughout therapy (which is universal standard of care) and it was assumed to impact all samples examined, therefore this antibiotic was not included in this study under ‘any antibiotic use’. We also know from previous research (Dunn et al., 2022) that these children are exposed to repeated long durations of antifungal agents that change the microbiome community. The impact of extended antifungal exposure on the taxa and its impact on ARGs was not addressed in this study and should be studied further especially the impact of the peptide antifungal agents on the peptide ARGs.

## 5 Conclusion

In summary, in this cohort of children and adolescents with leukemia/lymphoma we found that repeated courses and longer duration of antibiotics commonly given during periods of febrile neutropenia was associated with an increase in pathogenic bacteria and a decrease in “good” bacteria in the microbiome. This is important since the changes in the microbiome with repetitive long courses of broad-spectrum antibiotics are potentially creating an environment that favors ARGs and their associated pathogenic infections. The concern for this population is that increased ARGs in the GIT resistome coincide with a dysregulated immune environment and weakened gut epithelium, and this increases the likelihood of antibiotic-resistant life-threatening bacteremias, which increases morbidity and mortality in this population.

Further studies are needed to evaluate the clinical and epidemiological effects of long duration use of empiric broad-spectrum antibiotics on the resistome and on breakthrough infections with resistant organisms, as well as to evaluate the appropriate duration of empiric antibiotic treatment. Future efforts should also look at the impact of administering probiotics and different nutrition strategies in this population in an attempt to restore the “good” bacteria in the microbiome affected so drastically by antibiotic use. Antimicrobial stewardship program to reduce antibiotic overuse remains essential in this vulnerable population and should be considered in all decisions regarding antibiotic use.

## Data availability statement

The data presented in the study are deposited in the European Nucleotide Archive (https://www.ebi.ac.uk.ena), under accession numbers PRJEB29237, PRJEB41463, PRJEB46214, PRJEB53954, PRJEB59728.

## Ethics statement

The studies involving human participants were reviewed and approved by IWK Health Center Research Ethics Board (REB# 1019670 & 1022029). Written informed consent to participate in this study was provided by the participants’ legal guardian/next of kin.

## Author contributions

TM, KK and KD contributed to the conception and design of the study and interpretation of the results. KD and JM performed analyses. KD, JB, and TM wrote the manuscript. All authors secured funding for the project, contributed to manuscript revision, and read, and approved the submitted version.
