# Supplementary material for: The gastrointestinal antibiotic resistome in pediatric leukemia and lymphoma patients
Source: Front Cell Infect Microbiol. 2023 Feb 24;13:1102501. doi: 10.3389/fcimb.2023.1102501 (PMC9998685; doi:10.3389/fcimb.2023.1102501)
Supplement: Supplementary file 1 [file DataSheet_1.docx]

**Cancer types and subtypes of patients**

**Acute lymphoblastic leukemia** 28 patients

pre-B cell ALL (27)

T-cell ALL (1)

**Acute myloid leukemia**  5 patients

**Hodgkin’s lymphom**a 2 patients

Nodular sclerosis (2)

**Non-hodgkin’s lymphoma** 4 patients

Lymphoblastic lymphoma (1)

ALCL anaplastic large cell lymphoma (1)

Burkitt’s lymphoma (1)

Mediastinal B-cell lymphoma (1)

**Cancer treatment protocol details:**

***Acute Lymphoblastic Leukemia (ALL):***

*AALL0932:* Standard risk (SR) B-cell ALL that consists of Induction, consolidation, interim maintenance I, delayed intensification and interim maintenance II followed by 2 years from IM#I of maintenance therapy for girls and 3 years for boys.

-Induction: 4 weeks of treatment with corticosteroids, vincristine, pegaspargase, and intrathecal cytarabine and methotrexate.

-Consolidation: 4 weeks of treatment with mercaptopurine, vincristine and intrathecal methotrexate.

-Interim maintenance I & II: 8 weeks of treatment with low dose methotrexate, vincristine and intrathecal methotrexate.

-Delayed intensification: 8 weeks of treatment with doxorubincin, cytarabine, corticosteroids, pegaspargase, cyclophosphamide, thioguanine,

and intrathecal methotrexate.

-Maintenance: mercaptopurine, oral methotrexate, vincristine, corticosteroids and intrathecal methotrexate.

*AALL1131*: High risk (HR) and Very high risk (VHR) B-cell ALL that consists of Induction, consolidation, interim maintenance I, delayed intensification and interim maintenance II (VHR) followed by 2 years from IMI of maintenance therapy for girls and 3 years for boys.

-Induction: 4 weeks of treatment with corticosteroids, vincristine, pegaspargase, daunorubicin and intrathecal cytarabine and methotrexate.

-Consolidation: 8 weeks of treatment with mercaptopurine, vincristine, cyclophosphamide, cytarabine, pegaspargase and intrathecal methotrexate.

-Interim maintenance I: 8 weeks of treatment with high dose methotrexate, vincristine, mercaptopurine and intrathecal methotrexate.

-Delayed intensification: 8 weeks of treatment with doxorubincin, cytarabine, corticosteroids, pegaspargase, cyclophosphamide, thioguanine, and intrathecal methotrexate.

-Interim maintenance II: 8 weeks of treatment with low dose methotrexate, pegaspargase, vincristine and intrathecal methotrexate.

-Maintenance: mercaptopurine, oral methotrexate, vincristine, corticosteroids and intrathecal methotrexate.

*AALL15P1*: Infant ALL that consists of Induction, consolidation, interim maintenance, delayed intensification and 1.5 years of maintenance.

-Induction: 4 weeks of treatment with corticosteroids, vincristine, pegaspargase, cytarabine, daunorubicin and intrathecal cytarabine and methotrexate.

-Consolidation: 4 weeks of treatment with cyclophosphamide, cytarabine, and intrathecal methotrexate and cytarabine.

-Interim maintenance I: 4 weeks of treatment with high dose methotrexate, high dose cytarabine, vincristine, mercaptopurine, pegaspargase and intrathecal methotrexate and cytarabine.

-Delayed intensification: 8 weeks of treatment with daunorubicin, cytarabine, vincristine, corticosteroids, pegaspargase, cyclophosphamide, thioguanine and intrathecal cytarabine.

-Maintenance: mercaptopurine, vincristine, corticosteroids and intrathecal methotrexate and cytarabine.

*AALL0434*: T-cell acute lymphoblastic leukemia that consists of Induction, consolidation, interim maintenance I, delayed intensification followed by 2 years from IMI of maintenance therapy for girls and 3 years for boys. This includes nelarabine for intermediate and high risk patients given in consolidation, delayed intensification and the first 3 maintenance cycles.

-Induction: 4 weeks of treatment with corticosteroids, vincristine, pegaspargase, daunorubicin and intrathecal cytarabine and methotrexate.

-Consolidation: 8 weeks of treatment with mercaptopurine, vincristine, cyclophosphamide, cytarabine, pegaspargase and intrathecal methotrexate.

-Interim maintenance II: 8 weeks of treatment with low dose methotrexate, pegaspargase, vincristine and intrathecal methotrexate.

-Delayed intensification: 8 weeks of treatment with doxorubincin, cytarabine, corticosteroids, pegaspargase, cyclophosphamide, thioguanine, and intrathecal methotrexate.

-Maintenance: mercaptopurine, oral methotrexate, vincristine, corticosteroids and intrathecal methotrexate.

***Acute myeloid leukemia (AML):***

*AAML0531:* 6-8 months of therapy

-Induction I: cytarabine, daunorubicin and etoposide plus gemtuzumab with intrathecal cytarabine

-Induction II: cytarabine, daunorubicin and etoposide with intrathecal cytarabine

-Intensification I: high dose cytarabine and etoposide with intrathecal cytarabine

-Intensification II: mitoxantrone and cytarabine

-Intensification III: high dose cytarabine and erwinia asparaginase

*AAML1531:* Acute myeloid leukemia treatment for children with Down’s syndrome

-Induction I: cytarabine, daunorubicin and thioguanine with intrathecal cytarabine

-Induction II: mitoxantrone and etoposide

-Intensification I: high dose cytarabine and etoposide

-Intensification II: high dose cytarabine and erwinia asparaginase

***Hodgkin’s Lymphoma (HL)***: therapy consists of 2 to 4 cycles of ABVE-PC +/- radiation.

*AHOD0031:* Hodgkins Lymphoma for intermediate risk patients lasts for 2 to 4 months. The treatment is with doxorubicin, etoposide, cyclophosphamide, vincristine, bleomycin and prednisone cycling every 3 weeks.

***Non-Hodgkin’s Lymphoma (NHL):***

*AALL1231:* Treatment for T-cell lymphoblastic lymphoma with or without bortezomib.

-Induction: 4 weeks of treatment with corticosteroids, vincristine, pegaspargase, daunorubicin and intrathecal cytarabine and methotrexate.

-Consolidation: 8 weeks of treatment with mercaptopurine, vincristine, cyclophosphamide, cytarabine, pegaspargase and intrathecal methotrexate.

-Interim maintenance: 8 weeks of treatment with low dose methotrexate, pegaspargase, vincristine and intrathecal methotrexate.

-Delayed intensification: 8 weeks of treatment with doxorubincin, cytarabine, corticosteroids, pegaspargase, cyclophosphamide, thioguanine, and intrathecal methotrexate.

-Maintenance: mercaptopurine, oral methotrexate, vincristine, corticosteroids and intrathecal methotrexate.

*ALCL-99*: Treatment for anaplastic large cell lymphoma consists of 6 rotating cycles of 2 courses of chemotherapy.

-Course A: ifosfamide, corticosteroid, high dose methotrexate, etoposide and cytarabine.

-Course B: high dose methotrexate, doxorubicin, corticosteroid and cyclophosphamide.

*ANHL1131:* B-cell Non-Hodgkin Lymphoma or Mature B-cell includes treatment with rituximab for CD20 positive patients.

-COP: cyclophosphamide, vincristine, corticosteroid with intrathecal methotrexate and cytarabine

-COPADM#1 and 2: doxorubicin, cyclophosphamide, vincristine, high dose methotrexate, prednisone and intrathecal methotrexate and cytarabine.

-CYVE#1 and 2: high dose cytarabine, etoposide, cytarabine, high dose methotrexate and intrathecal methotrexate and cytarabine.

-Maintenance #1: doxorubicin, cyclophosphamide, vincristine, high dose methotrexate, prednisone and intrathecal methotrexate and cytarabine.

-Maintenance #2: cytarabine and etoposide.

*ANHL1131:* Primary mediastinal B cell lymphoma consists of 6 cycles of EPOCH that includes etoposide, cyclophosphamide, vincristine and doxorubicin, prednisone, rituximab (for CD20 positive patients) and intrathecal methotrexate.

**Supplemental Figure S1** Timelines of stool sample collection and antibiotic use for patients. Time is recorded from start of chemotherapy for both stool sample collection and antibiotic use. Antibiotic listed by antibiotic class.

**Supplemental Figure S2.** Distribution of the 141 metagenome species in the 127 stools samples, showing cancer type of samples and days from start of chemotherapy for each sample.

**Supplemental Table S1**. Antibiotics used in patient population and the class they belong to.

| Antibiotic | Antibiotic class | method of administration |
| --- | --- | --- |
| Piperacillin-tazobactam | β-lactam | IV |
| Amoxicillin and clavulanate | β-lactam | PO*/IV |
| Cloxacillin | β-lactam | IV |
| Ampicillin | β-lactam | PO |
| Ceftriaxone | β-lactam | IV |
| Cefazolin | β-lactam | IV |
| Ceftazidime | β-lactam | IV |
| Cephalexin | β-lactam | PO |
| Cefixime | β-lactam | PO |
| Cefotaxime | β-lactam | IV |
| Meropenem | β-lactam | IV |
| Vancomycin | glycopeptide/peptide | IV*/PO |
| Ciprofloxacin | fluoroquinolone | IV |
| Levofloxacin | fluoroquinolone | PO*/IV |
| Metronidazole | nitroimidazole | PO*/IV |
| Azithromycin | macrolide | IV*/PO |
| Clarithromycin | macrolide | PO |
| Tobramycin | aminoglycoside | IV |
| Clindamycin | lincosamide | IV |

*main method of administration

**Supplemental Table S2**. Number of patients and samples as well as cancer type used to create contiguous sequence fragments to identify genes to compare with the Comprehensive Antibiotic Resistance Database to identify antibiotic resistance genes. Accession Numbers are from ENA Projects: PRJEB46214, PRJEB53954, PRJEB59728

| Cancer type | Number of patients | Number of samples | Accession Numbers |
| --- | --- | --- | --- |
| acute lymphoblastic leukemia | 7 | 9 | ERR6281486, ERR6281487, ERR6281490, ERR6281492, ERR9922173, ERR6281494, ERR6281495, ERR6281496, ERS14606266 |
| acute myeloid leukemia | 5 | 18 | ERR9922148, ERR9922150, ERR9922151, ERR9922152, ERR9922153, ERR9922154, ERR9922155, ERR9922156, ERR9922158, ERR9922159, ERR9922160, ERR9922162, ERR9922164, ERR9922165, ERR9922166, ERR9922167, ERR9922168, ERS14606264 |
| hodgkin’s lymphoma | 2 | 2 | ERR9922144, ERR9922169 |
| non-hodgkin’s lymphoma | 5 | 8 | ERR9922146, ERR9922147, ERR6281484, ERR6281485, ERR9922171, ERR9922178, ERR9922179, ERR9922181 |
| neuroblastoma | 3 | 6 | ERS14606254, ERS14606255, ERS14606256, ERS14606262, ERS14606263, ERS14606265 |
| bone sarcoma | 1 | 1 | ERS14606261 |
| soft sarcoma | 1 | 4 | ERS14606257, ERS14606258, ERS14606259, ERS14606260 |
| total | 24 | 48 |  |

Supplemental Table S3 Patient and samples used in analyses of ARGs for multi-use/courses of β-lactams, vancomycin and AAb and duration/days of β-lactams and vancomycin after reduction for sample uniformity. Accession Numbers are from ENA Projects: PRJEB29237, PRJEB41463, PRJEB46214, PRJEB53954, PRJEB59728.

| Sample ID | β-lactam multiuse/courses dataset | vancomycin multiuse/courses dataset | AAb multiuse/courses dataset | β-lactam duration/days dataset | vancomycin duration dataset | Accession # |
| --- | --- | --- | --- | --- | --- | --- |
| MALL-001-ST01 | 1-2 courses | 1-2 courses | 1-2 courses | short | short | ERR2849623 |
| MALL-001-ST02 | 1-2 courses | 1-2 courses | 1-2 courses | short | short | ERR2849638 |
| MALL-001-ST03 | 1-2 courses | 3+ courses | 3+ courses | LM | short | ERR9922133 |
| MALL-002-ST01 | 1-2 courses | no use | 1-2 courses | short | no use | ERR2849624 |
| MALL-003-ST03 | 3+ courses | 3+ courses | 3+ courses | LM | short | ERR9922134 |
| MALL-004-ST01 | 1-2 courses | no use | 1-2 courses | short | no use | ERR2849625 |
| MALL-005-ST01 | 1-2 courses | 1-2 courses | 1-2 courses | short | short | ERR2849626 |
| MALL-006-ST01 | 3+ courses | 1-2 courses | 3+ courses | short | short | ERR2849627 |
| MALL-007-ST01 | 3+ courses | no use | 3+ courses | short | no use | ERR2849628 |
| MALL-007-ST02 | 3+ courses | 3+ courses | 3+ courses | LM | LM | ERR9922132 |
| MALL-008-ST01 | 1-2 courses | 1-2 courses | 3+ courses | short | short | ERR2849629 |
| MALL-008-ST02 | 3+ courses | 3+ courses | 3+ courses | short | short | ERR2849639 |
| MALL-008-ST03 | 3+ courses | 3+ courses | 3+ courses | LM | short | ERR2849647 |
| MALL-010-ST01 |  |  |  |  |  | ERR2849630 |
| MALL-010-ST02 |  |  |  |  |  | ERR2849640 |
| MALL-010-ST03 |  |  |  |  |  | ERR2849648 |
| MALL-010-ST04 |  |  |  |  |  | ERR2849654 |
| MALL-010-ST05 |  |  |  |  |  | ERR2849659 |
| MALL-010-ST06 |  |  |  |  |  | ERR6281450 |
| MALL-010-ST08 |  |  |  |  |  | ERR9922136 |
| MALL-010-ST10 |  |  |  |  |  | ERR6281452 |
| MALL-010-ST11 |  |  |  |  |  | ERS14606248 |
| MALL-010-ST12 |  |  |  |  |  | ERR4866072 |
| MALL-010-ST13 |  |  |  |  |  | ERS14606249 |
| MALL-010-ST14 |  |  |  |  |  | ERR9922137 |
| MALL-011-ST01 | 3+ courses | 1-2 courses | 3+ courses | LM | short | ERS14606250 |
| MALL-011-ST02 | 3+ courses | 3+ courses | 3+ courses | LM | short | ERS14606251 |
| MALL-011-ST03 | 3+ courses | 3+ courses | 3+ courses | LM | short | ERS14606252 |
| MALL-011-ST04 | 3+ courses | 3+ courses | 3+ courses | LM | LM | ERS14606253 |
| MALL-013-ST01 | 1-2 courses | 1-2 courses | 3+ courses | LM | short | ERR2849632 |
| MALL-013-ST02 | 1-2 courses | 1-2 courses | 3+ courses | LM | short | ERR2849642 |
| MALL-013-ST03 | 1-2 courses | 1-2 courses | 3+ courses | LM | short | ERR2849650 |
| MALL-013-ST04 | 3+ courses | 3+ courses | 3+ courses | LM | short | ERR2849655 |
| MALL-013-ST05 | 3+ courses | 3+ courses | 3+ courses | LM | short | ERR6281455 |
| MALL-013-ST06 | 3+ courses | 3+ courses | 3+ courses | LM | short | ERR6281456 |
| MALL-013-ST07 | 3+ courses | 3+ courses | 3+ courses | LM | short | ERR6281457 |
| MALL-014-ST01 | no use | no use | no use | no use | no use | ERR2849633 |
| MALL-014-ST02 | 1-2 courses | no use | 1-2 courses | short | no use | ERR2849643 |
| MALL-014-ST03 | 1-2 courses | no use | 1-2 courses | short | no use | ERR2849651 |
| MALL-014-ST04 | 3+ courses | no use | 3+ courses | short | no use | ERR9922135 |
| MALL-015-ST01 | 1-2 courses | no use | 1-2 courses | short | no use | ERR2849634 |
| MALL-015-ST04 | 3+ courses | 1-2 courses | 3+ courses | LM | short | ERR2849656 |
| MALL-016-ST01 |  |  | 1-2 courses | short |  | ERR2849635 |
| MALL-016-ST02 |  |  | 1-2 courses | short |  | ERR2849644 |
| MALL-016-ST05 |  |  | 1-2 courses | short |  | ERR2849652 |
| MALL-016-ST07 |  |  | 1-2 courses | short |  | ERR4866068 |
| MALL-016-ST08 |  |  | 1-2 courses | short |  | ERR6281458 |
| MALL-016-ST09 |  |  | 1-2 courses | short |  | ERR6281459 |
| MALL-016-ST10 |  |  | 1-2 courses | short |  | ERR6281460 |
| MALL-016-ST11 |  |  | 1-2 courses | short |  | ERR6281461 |
| MALL-016-ST12 |  |  | 1-2 courses | short |  | ERR6281462 |
| MALL-016-ST13 |  |  | 3+ courses | LM |  | ERR9922138 |
| MALL-016-ST14 |  |  | 3+ courses | LM |  | ERR6281464 |
| MALL-017-ST01 | 1-2 courses | no use | 1-2 courses | short | no use | ERR2849636 |
| MALL-017-ST02 | 1-2 courses | no use | 1-2 courses | short | no use | ERR2849645 |
| MALL-017-ST03 | 1-2 courses | 1-2 courses | 1-2 courses | short | short | ERR2849653 |
| MALL-017-ST04 | 1-2 courses | 1-2 courses | 1-2 courses | short | short | ERR2849658 |
| MALL-017-ST05 | 1-2 courses | 1-2 courses | 1-2 courses | short | short | ERR6281465 |
| MALL-018-ST01 | 1-2 courses | no use | 1-2 courses | short | no use | ERR2849637 |
| MALL-018-ST02 | 1-2 courses | no use | 1-2 courses | short | no use | ERR2849646 |
| MALL-019-ST01 | 1-2 courses | no use | 1-2 courses | short | no use | ERR6281466 |
| MALL-019-ST02 | 1-2 courses | no use | 1-2 courses | short | no use | ERR6281467 |
| MALL-019-ST03 | 1-2 courses | no use | 1-2 courses | short | no use | ERR9922139 |
| MALL-019-ST04 | 1-2 courses | no use | 1-2 courses | short | no use | ERR6281469 |
| MALL-020-ST01 | no use | no use | no use | no use | no use | ERR6281470 |
| MALL-020-ST02 | no use | no use | no use | no use | no use | ERR9922140 |
| MALL-020-ST03 | 1-2 courses | 1-2 courses |  |  |  | ERR9922141 |
| MALL-020-ST04 | 1-2 courses | 1-2 courses |  |  |  | ERR9922142 |
| MALL-020-ST05 | 1-2 courses | 1-2 courses |  |  |  | ERR6281474 |
| MALL-020-ST06 | 1-2 courses | 1-2 courses |  |  |  | ERR6281475 |
| MALL-020-ST07 | 1-2 courses | 1-2 courses |  |  |  | ERR6281476 |
| MALL-020-ST08 | 3+ courses | 1-2 courses |  |  |  | ERR9922143 |
| MALL-020-ST09 | 3+ courses | 1-2 courses |  |  |  | ERR6281478 |
| MALL-020-ST10 | 3+ courses | 3+ courses |  |  |  | ERR6281479 |
| MALL-020-ST11 | 3+ courses | 3+ courses |  |  |  | ERR6281480 |
| MALL-021-ST01 | 1-2 courses | no use | 3+ courses | short | no use | ERR6281481 |
| MALL-021-ST02 | 1-2 courses | no use | 3+ courses | short | no use | ERR6281482 |
| MALL-022-ST01 | no use | no use | no use | no use | no use | ERR6281483 |
| MONC-004-ST01 | no use | no use | no use | no use | no use | ERR9922144 |
| MONC-004-ST02 | 1-2 courses | no use | 1-2 courses | short | no use | ERR9922145 |
| MONC-005-ST01 | 1-2 courses | no use | 1-2 courses | short | no use | ERR9922146 |
| MONC-005-ST02 | 1-2 courses | no use | 1-2 courses | short | no use | ERR9922147 |
| MONC-007-ST01 | 1-2 courses | no use | 1-2 courses | short | no use | ERR9922148 |
| MONC-007-ST02 | 1-2 courses | no use | 1-2 courses | short | no use | ERR9922149 |
| MONC-007-ST03 | 1-2 courses | 1-2 courses | 3+ courses | LM | short | ERR9922150 |
| MONC-007-ST04 | 3+ courses | 1-2 courses | 3+ courses | LM | short | ERR9922151 |
| MONC-007-ST05 | 3+ courses | 3+ courses | 3+ courses | LM | short | ERR9922152 |
| MONC-007-ST06 | 3+ courses | 3+ courses | 3+ courses | LM | LM | ERR9922153 |
| MONC-008-ST01 | no use | no use | no use | no use | no use | ERR6281484 |
| MONC-008-ST02 | 1-2 courses | no use | 1-2 courses | short | no use | ERR6281485 |
| MONC-010-ST01 | 1-2 courses | 1-2 courses | 1-2 courses | LM | short | ERR9922154 |
| MONC-010-ST02 | 1-2 courses | 1-2 courses | 3+ courses | LM | LM | ERR9922155 |
| MONC-011-ST01 | 1-2 courses | no use | 1-2 courses | short | no use | ERR9922156 |
| MONC-011-ST02 | 1-2 courses | 1-2 courses |  |  |  | ERR9922157 |
| MONC-011-ST03 | 1-2 courses | 1-2 courses |  |  |  | ERR9922158 |
| MONC-011-ST04 | 3+ courses | 1-2 courses |  |  |  | ERR9922159 |
| MONC-011-ST05 | 3+ courses | 1-2 courses |  |  |  | ERR9922160 |
| MONC-011-ST06 | 3+ courses | 3+ courses |  |  |  | ERR9922161 |
| MONC-011-ST07 | 3+ courses | 3+ courses |  |  |  | ERR9922162 |
| MONC-011-ST08 | 3+ courses | 3+ courses |  |  |  | ERR9922163 |
| MONC-011-ST09 | 3+ courses | 3+ courses |  |  |  | ERR9922164 |
| MONC-011-ST10 | 3+ courses | 3+ courses |  |  |  | ERR9922165 |
| MONC-011-ST11 | 3+ courses | 3+ courses |  |  |  | ERR9922166 |
| MONC-013-ST01 | 1-2 courses | 1-2 courses | 1-2 courses | short | short | ERR9922167 |
| MONC-013-ST02 | 1-2 courses | 1-2 courses | 3+ courses | short | short | ERR9922168 |
| MONC-014-ST01 | 1-2 courses | no use | 1-2 courses | short | no use | ERR9922169 |
| MONC-015-ST01 | no use | no use | no use | no use | no use | ERR6281486 |
| MONC-016-ST01 | 1-2 courses | 1-2 courses | 1-2 courses | short | short | ERR9922170 |
| MONC-016-ST02 | 1-2 courses | 1-2 courses | 3+ courses | LM | short | ERS14606264 |
| MONC-017-ST01 | 1-2 courses | no use | 1-2 courses | short | no use | ERR9922171 |
| MONC-018-ST01 | 1-2 courses | no use | 1-2 courses | LM | no use | ERR6281487 |
| MONC-018-ST02 | 1-2 courses | no use | 1-2 courses | LM | no use | ERR6281488 |
| MONC-022-ST01 | 1-2 courses | no use | 1-2 courses | short | no use | ERR9922172 |
| MONC-022-ST02 | 1-2 courses | no use | 1-2 courses | short | no use | ERR6281490 |
| MONC-022-ST03 | 3+ courses | no use | 3+ courses | short | no use | ERR6281491 |
| MONC-022-ST04 | 3+ courses | no use | 3+ courses | short | no use | ERR6281492 |
| MONC-022-ST05 | 3+ courses | no use | 3+ courses | short | no use | ERR9922173 |
| MONC-025-ST01 | no use | no use | no use | no use | no use | ERR6281493 |
| MONC-025-ST02 | no use | no use | no use | no use | no use | ERR6281494 |
| MONC-026-ST01 | 1-2 courses | no use | 1-2 courses | short | no use | ERR6281495 |
| MONC-026-ST02 | 3+ courses | 1-2 courses | 3+ courses | short | short | ERR9922175 |
| MONC-027-ST01 | 1-2 courses | no use | 1-2 courses | short | no use | ERR6281496 |
| MONC-027-ST02 | 1-2 courses | no use | 1-2 courses | short | no use | ERR9922176 |
| MONC-030-ST01 | 1-2 courses | no use | 1-2 courses | short | no use | ERR9922178 |
| MONC-030-ST02 | 3+ courses | 1-2 courses | 3+ courses | short | short | ERR9922179 |
| MONC-032-ST01 | no use | no use | no use | no use | no use | ERR9922181 |
| MONC-033-ST01 | 1-2 courses | no use | 1-2 courses | short | no use | ERR6281498 |

**Supplemental Table S4** Results of comparisons of species richness and evenness with repeated courses of β-lactams, vancomycin and AAb and duration of β-lactams and vancomycin.

|  | β-lactam | | vancomycin | | AAb | |
| --- | --- | --- | --- | --- | --- | --- |
| repeated use | **richness** | **evenness** | **richness** | **evenness** | **richness** | **evenness** |
| p value^a^ | **8.23e-5** | 0.127 | **0.047** | 0.992 | **1.3e-4** | 0.059 |
| no use vs 1-2^b^ | 0.208 | na | 0.248 | na | 0.337 | na |
| no use vs 3+ ^b^ | **9.0e-4** | na | 0.052 | na | **0.003** | na |
| 1-2 vs 3+ ^b^ | **7.1e-4** | na | 0.544 | na | **0.001** | na |
|  |  |  |  |  |  |  |
| duration |  |  |  |  |  |  |
| p value^a^ | **0.005** | 0.076 | 0.082 | 0.998 | na | na |
| no use vs short^b^ | 0.171 | na | na | na |  |  |
| no use vs LM ^b^ | **0.009** | na | na | na |  |  |
| short vs LM ^b^ | 0.061 | na | na | na |  |  |

^a^ ANOVA p value; ^b^ post hoc pairwise analysis using Tukey’s honest significance difference test, adjusted for multiple tests.

**Supplemental Table S5** Results of ALDEx2 Kruskal-Wallis test corrected for false discovery (BH) of bacterial phyla with repeated course of β -lactams, vancomycin and any antibiotic as well as increased duration of β -lactams and vancomycin.

|  | Bacteroidetes | Firmicutes | Proteobacteria | Actinobacteria | Fusobacteria | Verrucomicrobia |
| --- | --- | --- | --- | --- | --- | --- |
| repeated courses |  |  |  |  |  |  |
| β -lactams | **1.8e-4 D** | **0.0053 I** | **7.3e-7 I** | 0.2942 | 0.0670 | 0.6582 |
| vancomycin | **0.0120 D** | 0.1079 | **2.0e-4 I** | 0.1109 | **0.0486 I** | 0.3288 |
| any antibiotic | **4.5e-7 D** | **0.0035 I** | **5.1e-5 I** | 0.5753 | **0.0199 I** | 0.6985 |
| duration |  |  |  |  |  |  |
| β -lactams | **5.2e-4 D** | 0.0938 | **5.7e-4 I** | 0.6654 | **0.0067 I** | 0.7422 |
| vancomycin | 0.3197 | 0.2254 | **0.0191 I** | 0.1592 | 0.1179 | 0.4768 |

D=relative abundance decreased; I=relative abundance increased

**Supplemental Table S6** List of CARD genes with ≥ 60% sequence identity in samples and the antibiotic class and mechanism, including number of the 127 samples that contained the gene.

| Gene | Antibiotic class | Resistance Mechanism | Number samples with gene (N=127) |
| --- | --- | --- | --- |
| gyrB conferring resistance to fluoroquinolones | fluoroquinolone | antibiotic target alteration | 127 |
| rpoB | rifamycin | antibiotic target alteration; antibiotic target replacement | 127 |
| EF-Tu mutants conferring resistance to kirromycin | elfamycin | antibiotic target alteration | 125 |
| EF-Tu mutants conferring resistance to Pulvomycin | elfamycin | antibiotic target alteration | 125 |
| rpoB2 | rifamycin | antibiotic target alteration; antibiotic target replacement | 124 |
| efrA | multidrug | antibiotic efflux | 123 |
| fusA with mutation conferring resistance to fusidic acid | fusidic acid | antibiotic target alteration | 123 |
| ugd | peptide | antibiotic target alteration | 120 |
| tet(W/N/W) | tetracycline | antibiotic target protection | 116 |
| thyA with mutation conferring resistance to para-aminosalicylic acid | para-aminosalicylic acid | antibiotic target alteration | 114 |
| efrB | multidrug | antibiotic efflux | 112 |
| gyrA conferring resistance to fluoroquinolones | fluoroquinolone | antibiotic target alteration | 112 |
| LlmA 23S ribosomal RNA methyltransferase | lincosamide | antibiotic target alteration | 111 |
| mel | multidrug | antibiotic target protection | 111 |
| EF-Tu mutants conferring resistance to GE2270A | elfamycin | antibiotic target alteration | 110 |
| msrE | multidrug | antibiotic target protection | 106 |
| tetO | tetracycline | antibiotic target protection | 103 |
| patA | fluoroquinolone | antibiotic efflux | 102 |
| vanRG | glycopeptide | antibiotic target alteration | 101 |
| patB | fluoroquinolone | antibiotic efflux | 99 |
| rpsL mutations conferring resistance to Streptomycin | aminoglycoside | antibiotic target alteration | 99 |
| vanRA | glycopeptide | antibiotic target alteration | 93 |
| msbA | nitroimidazole | antibiotic efflux | 92 |
| rosA | peptide | antibiotic efflux | 89 |
| ileS conferring resistance to mupirocin | mupirocin | antibiotic target alteration | 88 |
| lsaB | multidrug | antibiotic target protection | 88 |
| tet37 | tetracycline | antibiotic inactivation | 88 |
| vanRI | glycopeptide | antibiotic target alteration | 87 |
| tet32 | tetracycline | antibiotic target protection | 86 |
| ANT(6)-Ib | aminoglycoside | antibiotic inactivation | 85 |
| pmrA | fluoroquinolone | antibiotic efflux | 81 |
| acrD | aminoglycoside | antibiotic efflux | 80 |
| mupB conferring resistance to mupirocin | mupirocin | antibiotic target alteration | 80 |
| RlmA(II) | multidrug | antibiotic target alteration | 80 |
| tetQ | tetracycline | antibiotic target protection | 80 |
| PBP2x conferring resistance to amoxicillin | ß-lactam | antibiotic target alteration | 78 |
| tetM | tetracycline | antibiotic target protection | 76 |
| OmpK37 | ß-lactam | reduced permeability to antibiotic | 75 |
| acrB | multidrug | antibiotic efflux | 73 |
| PBP1a conferring resistance to amoxicillin | ß-lactam | antibiotic target alteration | 72 |
| vanRD | glycopeptide | antibiotic target alteration | 72 |
| tet(40) | tetracycline | antibiotic efflux | 71 |
| vanUG | glycopeptide | antibiotic target alteration | 71 |
| mdtB | aminocoumarin | antibiotic efflux | 68 |
| vanVB | glycopeptide | antibiotic target alteration | 68 |
| CfxA4 | ß-lactam | antibiotic inactivation | 66 |
| LptD | multidrug | antibiotic efflux | 65 |
| cepA | ß-lactam | antibiotic inactivation | 64 |
| cpxA | multidrug | antibiotic efflux | 64 |
| CRP | multidrug | antibiotic efflux | 64 |
| mdtG | fosfomycin | antibiotic efflux | 64 |
| ampH beta-lactamase | ß-lactam | antibiotic inactivation | 62 |
| eptB | peptide | antibiotic target alteration | 62 |
| TolC | multidrug | antibiotic efflux | 62 |
| AcrF | multidrug | antibiotic efflux | 61 |
| arnA | peptide | antibiotic target alteration | 61 |
| Mef(En2) | macrolide | antibiotic efflux | 61 |
| acrA | multidrug | antibiotic efflux | 60 |
| mdtC | aminocoumarin | antibiotic efflux | 60 |
| AcrE | multidrug | antibiotic efflux | 59 |
| acrR | multidrug | antibiotic target alteration; antibiotic efflux | 59 |
| TEM-1 | ß-lactam | antibiotic inactivation | 59 |
| OmpA | ß-lactam | reduced permeability to antibiotic | 58 |
| rosB | peptide | antibiotic efflux | 58 |
| UhpT with mutation conferring resistance to fosfomycin | fosfomycin | antibiotic target alteration | 58 |
| macB | macrolide | antibiotic efflux | 57 |
| APH(3')-IIIa | aminoglycoside | antibiotic inactivation | 56 |
| kdpE | aminoglycoside | antibiotic efflux | 56 |
| MexB | multidrug | antibiotic efflux | 56 |
| tetA(46) | tetracycline | antibiotic efflux | 56 |
| baeR | multidrug | antibiotic efflux | 55 |
| poxtA | multidrug | antibiotic target protection | 55 |
| sdiA | multidrug | antibiotic efflux | 55 |
| MdtK | fluoroquinolone | antibiotic efflux | 54 |
| soxR | multidrug | antibiotic target alteration; antibiotic efflux | 54 |
| eptA | peptide | antibiotic target alteration | 53 |
| PmrF | peptide | antibiotic target alteration | 53 |
| emrR | fluoroquinolone | antibiotic efflux | 52 |
| mdtN | multidrug | antibiotic efflux | 52 |
| lnuC | lincosamide | antibiotic inactivation | 51 |
| mdtA | aminocoumarin | antibiotic efflux | 51 |
| ErmG | multidrug | antibiotic target alteration | 50 |
| mdfA | multidrug | antibiotic efflux | 50 |
| tetB(P) | tetracycline | antibiotic target protection | 50 |
| CfxA6 | ß-lactam | antibiotic inactivation | 49 |
| CblA-1 | ß-lactam | antibiotic inactivation | 48 |
| H-NS | multidrug | antibiotic efflux | 48 |
| marA | multidrug | antibiotic efflux; reduced permeability to antibiotic | 48 |
| soxS with mutation conferring antibiotic resistance | multidrug | antibiotic target alteration; antibiotic efflux; reduced permeability to antibiotic | 48 |
| CBP-1 | ß-lactam | antibiotic inactivation | 47 |
| CfxA2 | ß-lactam | antibiotic inactivation | 47 |
| mdtF | multidrug | antibiotic efflux | 47 |
| PBP2b conferring resistance to amoxicillin | ß-lactam | antibiotic target alteration | 47 |
| adeF | multidrug | antibiotic efflux | 46 |
| KpnE | multidrug | antibiotic efflux | 46 |
| KpnF | multidrug | antibiotic efflux | 45 |
| mdtO | multidrug | antibiotic efflux | 45 |
| rsmA | multidrug | antibiotic efflux | 45 |
| emrB | fluoroquinolone | antibiotic efflux | 44 |
| lmrD | lincosamide | antibiotic efflux | 44 |
| ErmF | multidrug | antibiotic target alteration | 43 |
| mdtE | multidrug | antibiotic efflux | 43 |
| bacA | peptide | antibiotic target alteration | 42 |
| emrK | tetracycline | antibiotic efflux | 42 |
| evgA | multidrug | antibiotic efflux | 42 |
| ampC beta-lactamase | ß-lactam | antibiotic inactivation | 41 |
| evgS | multidrug | antibiotic efflux | 41 |
| mdtP | multidrug | antibiotic efflux | 41 |
| tetA(P) | tetracycline | antibiotic efflux | 41 |
| vatB | streptogramin | antibiotic inactivation | 41 |
| emrA | fluoroquinolone | antibiotic efflux | 40 |
| mdtM | multidrug | antibiotic efflux | 40 |
| RanA | aminoglycoside | antibiotic efflux | 40 |
| YojI | peptide | antibiotic efflux | 40 |
| gadX | multidrug | antibiotic efflux | 39 |
| ErmB | multidrug | antibiotic target alteration | 38 |
| gadW | multidrug | antibiotic efflux | 38 |
| KpnG | multidrug | antibiotic efflux | 38 |
| chloramphenicol acetyltransferase | phenicol | antibiotic inactivation | 37 |
| tetX | multidrug | antibiotic inactivation | 37 |
| ArnT | peptide | antibiotic target alteration | 35 |
| oqxA | multidrug | antibiotic efflux | 34 |
| ampC1 beta-lactamase | ß-lactam | antibiotic inactivation | 32 |
| GlpT with mutation conferring resistance to fosfomycin | fosfomycin | antibiotic target alteration | 32 |
| tetB(46) | tetracycline | antibiotic efflux | 32 |
| AAC(6')-Ie-APH(2'')-Ia | aminoglycoside | antibiotic inactivation | 31 |
| AcrS | multidrug | antibiotic efflux | 31 |
| baeS | multidrug | antibiotic efflux | 31 |
| novA | aminocoumarin | antibiotic efflux | 31 |
| tetA(60) | tetracycline | antibiotic efflux | 31 |
| vanSG | glycopeptide | antibiotic target alteration | 31 |
| fusE with mutation conferring resistance to fusidic acid | fusidic acid | antibiotic target alteration | 30 |
| vanYB | glycopeptide | antibiotic target alteration | 30 |
| eatAv | multidrug | antibiotic target protection | 29 |
| aadS | aminoglycoside | antibiotic inactivation | 28 |
| liaS mutant conferring daptomycin resistance | peptide | antibiotic target alteration; antibiotic efflux | 28 |
| PBP3 conferring resistance to beta-lactam antibiotics | ß-lactam | antibiotic target alteration | 28 |
| tet(44) | tetracycline | antibiotic target protection | 28 |
| tetB(60) | tetracycline | antibiotic efflux | 28 |
| dfrF | diaminopyrimidine | antibiotic target replacement | 27 |
| vatE | streptogramin | antibiotic inactivation | 27 |
| lsaC | multidrug | antibiotic target protection | 26 |
| sul2 | sulfonamide | antibiotic target replacement | 26 |
| hmrM | multidrug | antibiotic efflux | 25 |
| ramA | multidrug | antibiotic efflux; reduced permeability to antibiotic | 25 |
| lsaA | multidrug | antibiotic target protection | 24 |
| Tet(X6) | tetracycline | antibiotic inactivation | 24 |
| ANT(9)-Ia | aminoglycoside | antibiotic inactivation | 23 |
| ErmQ | multidrug | antibiotic target alteration | 23 |
| fabI mutations conferring resistance to isoniazid and triclosan | multidrug | antibiotic target alteration | 22 |
| vanTG | glycopeptide | antibiotic target alteration | 22 |
| ACT-2 | ß-lactam | antibiotic inactivation | 21 |
| APH(3'')-Ib | aminoglycoside | antibiotic inactivation | 21 |
| RbpA | rifamycin | antibiotic target protection | 21 |
| Tet(X1) | tetracycline | antibiotic inactivation | 21 |
| msrC | multidrug | antibiotic target protection | 20 |
| vatH | streptogramin | antibiotic inactivation | 20 |
| AxyY | multidrug | antibiotic efflux | 19 |
| FosA5 | fosfomycin | antibiotic inactivation | 19 |
| LnuP | lincosamide | antibiotic inactivation | 19 |
| vanTC | glycopeptide | antibiotic target alteration | 19 |
| efmA | multidrug | antibiotic efflux | 18 |
| VatI | streptogramin | antibiotic inactivation | 18 |
| AAC(6')-Ii | aminoglycoside | antibiotic inactivation | 17 |
| APH(6)-Id | aminoglycoside | antibiotic inactivation | 17 |
| vanG | glycopeptide | antibiotic target alteration | 17 |
| emeA | acridine dye | antibiotic efflux | 16 |
| vanC | glycopeptide | antibiotic target alteration | 16 |
| vanSC | glycopeptide | antibiotic target alteration | 16 |
| FosA2 | fosfomycin | antibiotic inactivation | 15 |
| ramR mutants | multidrug | antibiotic target alteration; antibiotic efflux | 15 |
| vanWG | glycopeptide | antibiotic target alteration | 15 |
| catA4 | phenicol | antibiotic inactivation | 14 |
| ErmX | multidrug | antibiotic target alteration | 14 |
| vanRC | glycopeptide | antibiotic target alteration | 14 |
| vanXYC | glycopeptide | antibiotic target alteration | 14 |
| bcrA | peptide | antibiotic efflux | 13 |
| mphE | macrolide | antibiotic inactivation | 12 |
| MuxB | multidrug | antibiotic efflux | 12 |
| oqxB | multidrug | antibiotic efflux | 12 |
| AAC(6')-Im | aminoglycoside | antibiotic inactivation | 11 |
| LEN-32 | ß-lactam | antibiotic inactivation | 11 |
| mtrA | multidrug | antibiotic efflux | 11 |
| mtrD | multidrug | antibiotic efflux | 11 |
| rphB | rifamycin | antibiotic inactivation | 11 |
| ErmA | multidrug | antibiotic target alteration | 10 |
| mdsB | multidrug | antibiotic efflux | 10 |
| uL3 mutations conferring resistance to pleuromutilin antibiotics | pleuromutilin | antibiotic target alteration | 10 |
| APH(2'')-IIa | aminoglycoside | antibiotic inactivation | 9 |
| arlS | multidrug | antibiotic efflux | 9 |
| LpsA | peptide | reduced permeability to antibiotic | 9 |
| adeB | multidrug | antibiotic efflux | 8 |
| ParR | multidrug | antibiotic efflux; reduced permeability to antibiotic | 8 |
| smeB | multidrug | antibiotic efflux | 8 |
| tet(45) | tetracycline | antibiotic efflux | 8 |
| vanHD | glycopeptide | antibiotic target alteration | 8 |
| vanRF | glycopeptide | antibiotic target alteration | 8 |
| APH(2'')-IVa | aminoglycoside | antibiotic inactivation | 7 |
| optrA | multidrug | antibiotic target protection | 7 |
| vanRO | glycopeptide | antibiotic target alteration | 7 |
| cmeB | multidrug | antibiotic efflux | 6 |
| dfrG | diaminopyrimidine | antibiotic target replacement | 6 |
| golS | multidrug | antibiotic efflux | 6 |
| L1 beta-lactamase | ß-lactam | antibiotic inactivation | 6 |
| MexF | multidrug | antibiotic efflux | 6 |
| smeE | multidrug | antibiotic efflux | 6 |
| vanI | glycopeptide | antibiotic target alteration | 6 |
| catP | phenicol | antibiotic inactivation | 5 |
| cmx | phenicol | antibiotic efflux | 5 |
| mdsC | multidrug | antibiotic efflux | 5 |
| QnrB71 | fluoroquinolone | antibiotic target protection | 5 |
| rgt1438 | rifamycin | antibiotic inactivation | 5 |
| smeC | multidrug | antibiotic efflux | 5 |
| smeF | multidrug | antibiotic efflux | 5 |
| AAC(6')-Iad | aminoglycoside | antibiotic inactivation | 4 |
| APH(3')-VIIa | aminoglycoside | antibiotic inactivation | 4 |
| farB | antibacterial free fatty acids | antibiotic efflux | 4 |
| HERA-1 | ß-lactam | antibiotic inactivation | 4 |
| MCR-8.1 | peptide | antibiotic target alteration | 4 |
| mtrC | multidrug | antibiotic efflux | 4 |
| OXA-347 | ß-lactam | antibiotic inactivation | 4 |
| RanB | aminoglycoside | antibiotic efflux | 4 |
| smeD | multidrug | antibiotic efflux | 4 |
| smeR | multidrug | antibiotic efflux | 4 |
| smeS | multidrug | antibiotic efflux | 4 |
| tetU | tetracycline | antibiotic efflux | 4 |
| TxR | tetracycline | antibiotic efflux | 4 |
| AAC(6')-Ib8 | aminoglycoside | antibiotic inactivation | 3 |
| adeR | multidrug | antibiotic efflux | 3 |
| APH(3')-IIc | aminoglycoside | antibiotic inactivation | 3 |
| catB8 | phenicol | antibiotic inactivation | 3 |
| catI | phenicol | antibiotic inactivation | 3 |
| emrE | macrolide | antibiotic efflux | 3 |
| EreD | macrolide | antibiotic inactivation | 3 |
| ErmC | multidrug | antibiotic target alteration | 3 |
| macA | macrolide | antibiotic efflux | 3 |
| mdsA | multidrug | antibiotic efflux | 3 |
| vanL | glycopeptide | antibiotic target alteration | 3 |
| vanXD | glycopeptide | antibiotic target alteration | 3 |
| vatF | streptogramin | antibiotic inactivation | 3 |
| aadK | aminoglycoside | antibiotic inactivation | 2 |
| ACI-1 | ß-lactam | antibiotic inactivation | 2 |
| bmr | multidrug | antibiotic efflux | 2 |
| catB | phenicol | antibiotic inactivation | 2 |
| cfrC | multidrug | antibiotic target alteration | 2 |
| Erm(39) | multidrug | antibiotic target alteration | 2 |
| Erm(K) | multidrug | antibiotic target alteration | 2 |
| mecA | ß-lactam | antibiotic target replacement | 2 |
| msrA | multidrug | antibiotic target protection | 2 |
| qacA | fluoroquinolone | antibiotic efflux | 2 |
| smeA | multidrug | antibiotic efflux | 2 |
| tet(C) | tetracycline | antibiotic efflux | 2 |
| Tet(X3) | multidrug | antibiotic inactivation | 2 |
| ykkC | multidrug | antibiotic efflux | 2 |
| abeS | multidrug | antibiotic efflux | 1 |
| APH(2'')-Ig | aminoglycoside | antibiotic inactivation | 1 |
| apmA | aminoglycoside | antibiotic inactivation | 1 |
| FosB2 | fosfomycin | antibiotic inactivation | 1 |
| fusB | fusidic acid | antibiotic target protection | 1 |
| lnuG | lincosamide | antibiotic inactivation | 1 |
| mecR1 | ß-lactam | antibiotic target replacement | 1 |
| MexD | multidrug | antibiotic efflux | 1 |
| tetR | tetracycline | antibiotic target alteration; antibiotic efflux | 1 |
| vanRM | glycopeptide | antibiotic target alteration | 1 |

**Supplemental Table S7** Counts of genes per antibiotic class and number of significant genes (KW p_BH_ <0.05) when analyzed for the effects of repeated courses of antibiotics.

|  | Total | ß-lactam | Vancomycin | any antibiotic |
| --- | --- | --- | --- | --- |
| multidrug | 69 | 37 | 22 | 41 |
| glycopeptide antibiotic | 19 | 7 | 2 | 8 |
| ß-lactam | 18 | 9 | 4 | 10 |
| tetracycline antibiotic | 18 | 6 | 4 | 7 |
| aminoglycoside antibiotic | 15 | 7 | 3 | 7 |
| peptide antibiotic | 13 | 8 | 6 | 8 |
| fluoroquinolone antibiotic | 9 | 4 | 3 | 4 |
| fosfomycin | 5 | 3 | 3 | 3 |
| aminocoumarin antibiotic | 4 | 4 | 3 | 4 |
| lincosamide antibiotic | 4 | 1 | 0 | 2 |
| rifamycin antibiotic | 4 | 1 | 0 | 1 |
| streptogramin antibiotic | 4 | 0 | 0 | 1 |
| elfamycin antibiotic | 3 | 2 | 0 | 2 |
| macrolide antibiotic | 3 | 1 | 1 | 1 |
| mupirocin | 2 | 1 | 1 | 1 |
| fusidic acid | 2 | 0 | 0 | 1 |
| phenicol antibiotic | 2 | 0 | 0 | 0 |
| acridine dye | 1 | 1 | 1 | 1 |
| diaminopyrimidine antibiotic | 1 | 1 | 1 | 1 |
| nitroimidazole antibiotic | 1 | 1 | 1 | 1 |
| pleuromutilin antibiotic | 1 | 1 | 0 | 1 |
| para-aminosalicylic acid | 1 | 0 | 0 | 0 |
| sulfonamide antibiotic | 1 | 0 | 0 | 0 |
| Total | 200 | 95 | 55 | 105 |

**Supplemental Table S8**. Excel spreadsheet of results of linear models using LIMMA for repeated courses of ß-lactam, vancomycin or AAb (no use, 1-2 courses, 3+ courses) on ARGs from the ß-lactam, glycopeptide, peptide and multidrug antibiotic classes with cancer diagnosis and days from start of chemotherapy treated as confounders, and significance corrected for multiple tests using BH.

**Supplemental Table S9**. Excel spreadsheet of results of linear models using LIMMA for counts of courses of ß-lactam, vancomycin or AAb on ARGs from the ß-lactam, glycopeptide, peptide and multidrug antibiotic classes with cancer diagnosis and days from start of chemotherapy treated as confounders, and significance corrected for multiple tests using BH.

**Supplemental Table S10.** Excel spreadsheet of results of linear models using LIMMA for duration of ß-lactam or vancomycin (no use, short duration, LM duration) on ARGs from the ß-lactam, glycopeptide, peptide and multidrug antibiotic classes with cancer diagnosis and days from start of chemotherapy treated as confounders, and significance corrected for multiple tests using BH.

**Supplemental Table S11.** Excel spreadsheet of results of linear models using LIMMA for days of ß-lactam or vancomycin on ARGs from the ß-lactam, glycopeptide, peptide and multidrug antibiotic classes with cancer diagnosis and days from start of chemotherapy treated as confounders, and significance corrected for multiple tests using BH.

**Supplemental Table S12.** Excel spreadsheet of Spearman correlation analysis comparison of 141 metagenome species and the 78 ARGs were significant or candidate genes with repeated antibiotic courses and duration. Tabs include p-values, adjusted p-values using the method of Benjimini and Hochberg and Spearman rho values.
